# Evolution and diversity of *Rickettsia *bacteria

**DOI:** 10.1186/1741-7007-7-6

**Published:** 2009-02-02

**Authors:** Lucy A Weinert, John H Werren, Alexandre Aebi, Graham N Stone, Francis M Jiggins

**Affiliations:** 1 Institute of Evolutionary Biology, University of Edinburgh, Edinburgh, EH9 3JT, UK; 2 Biology Department, University of Rochester, Rochester, NY 14627, USA; 3 Agroscope Reckenholz-Tänikon, Research Station ART, Reckenholzstrasse 191, 8046 Zürich, Switzerland

## Abstract

**Background:**

*Rickettsia *are intracellular symbionts of eukaryotes that are best known for infecting and causing serious diseases in humans and other mammals. All known vertebrate-associated *Rickettsia *are vectored by arthropods as part of their life-cycle, and many other *Rickettsia *are found exclusively in arthropods with no known secondary host. However, little is known about the biology of these latter strains. Here, we have identified 20 new strains of *Rickettsia *from arthropods, and constructed a multi-gene phylogeny of the entire genus which includes these new strains.

**Results:**

We show that *Rickettsia *are primarily arthropod-associated bacteria, and identify several novel groups within the genus. *Rickettsia *do not co-speciate with their hosts but host shifts most often occur between related arthropods. *Rickettsia *have evolved adaptations including transmission through vertebrates and killing males in some arthropod hosts. We uncovered one case of horizontal gene transfer among *Rickettsia*, where a strain is a chimera from two distantly related groups, but multi-gene analysis indicates that different parts of the genome tend to share the same phylogeny.

**Conclusion:**

Approximately 150 million years ago, *Rickettsia *split into two main clades, one of which primarily infects arthropods, and the other infects a diverse range of protists, other eukaryotes and arthropods. There was then a rapid radiation about 50 million years ago, which coincided with the evolution of life history adaptations in a few branches of the phylogeny. Even though *Rickettsia *are thought to be primarily transmitted vertically, host associations are short lived with frequent switching to new host lineages. Recombination throughout the genus is generally uncommon, although there is evidence of horizontal gene transfer. A better understanding of the evolution of *Rickettsia *will help in the future to elucidate the mechanisms of pathogenicity, transmission and virulence.

## Background

*Rickettsia *bacteria are intracellular symbionts of eukaryotes. The genus is classified in the family Rickettsiaceae within the alpha-proteobacteria, and is closely related to the genera *Erlichia *and *Wolbachia *[1,2]. *Rickettsia *are most noted for causing human diseases, including Rocky Mountain spotted fever and epidemic typhus, which has been a major source of mortality at times in human history [3]. However, all known vertebrate-associated *Rickettsia *are vectored by arthropods as part of their life-cycle, and many other *Rickettsia *are found exclusively in arthropods with no known secondary host (for convenience, we will refer to the former as 'vertebrate *Rickettsia*' and the latter as 'arthropod *Rickettsia*'). In recent years, arthropod *Rickettsia *have been discovered in a diverse range of hosts, suggesting that they are more common than had been suspected [4-16]. Nevertheless, research effort has tended to concentrate on the medically important vertebrate *Rickettsia*, or on the more common arthropod endosymbionts, such as *Wolbachia *and *Cardinium*, and so we know little about the biology of arthropod *Rickettsia*. Even less is known about the closely related bacteria that have been recently discovered in organisms such as leeches and protists, and in metagenomic studies sequencing all DNA in an environmental sample [17-25]. This neglect is unfortunate, because comparing the vertebrate pathogens with related species can help to elucidate the mechanisms of pathogenicity, transmission and virulence [26,27]. However, this requires a robust phylogeny for the genus.

Historically, *Rickettsia *were classified into three major groups based on serological characteristics, namely the 'typhus group', 'spotted fever group' and 'scrub typhus group', although subsequent DNA sequencing led to the latter being reassigned to the related genus *Orientia *[28]. The relationship of species within the remaining two groups of *Rickettsia *has been the subject of intensive study over the last decade as progressively more informative genes have been sequenced [29-32] culminating in a multi-genic approach [33]. As a result it has been suggested that the spotted fever group consists of two sister clades, one of which is now designated 'transitional' [34] (although see [35]). A fourth so-called 'ancestral' clade, including *Rickettsia bellii *and *Rickettsia canadensis*, is thought to be basal to the other groups and is largely non-pathogenic to vertebrates. However, the position of *R. canadensis *remains uncertain [33].

While many studies have helped to clarify the relationships between the vertebrate *Rickettsia*, only one recent study has explored the relationship of the well classified groups to the newly discovered arthropod *Rickettsia *[36]. The authors found that most arthropod *Rickettsia *are basal to the vertebrate *Rickettsia *and that the *Rickettsia *associated with leeches, protists and freshwater environments fell into two phylogenetic groups, distinct from the arthropod and vertebrate groups. The only known exceptions are a small number of arthropod *Rickettsia *that fell within the group otherwise infecting leeches [12,36,37]. However, Perlman et al. [36] were only able to provide little statistically significant support for relationships among the arthropod *Rickettsia*. This is almost certainly because the study relied on partial sequences of *16S *rDNA, which is extremely slowly evolving, and so lacking in phylogenetic resolution. Improving this situation is challenging because amplifying other genes in basal strains has proven problematic, perhaps because the genes in question may either be missing or too divergent for PCR amplification using existing primers. Also, resolving some deep nodes in the *Rickettsia *species tree continues to be a problem. The reasons for this are unclear but could be exacerbated by long-branch attraction. One of the best ways to minimise this effect is to sample for more taxa and add them to the tree in the hope of breaking up (thereby shortening) the long branches.

Here, to explore the diversity of arthropod *Rickettsia*, we screened 4454 arthropods to uncover new *Rickettsia *strains and sequenced four genes from five known and 20 new bacterial strains. We use the recently published *Orientia tsutsugamushi *genome [38] to design PCR primers allowing amplification of rapidly evolving genes from strains that lie between the genera *Rickettsia *and *Orientia*. To include other hosts, we also searched published metagenomic databases for *Rickettsia *sequences. With these data, we have been able to produce the first well-resolved phylogeny of the entire genus *Rickettsia*, showing how the vertebrate *Rickettsia *relate to the other taxa. Our phylogeny has allowed us to identify and name additional novel groups. Furthermore, we were able to compare host associations among these groups, identify major life history transitions, and investigate the extent of recombination within the genus.

## Results

### Strains identified and genes sequenced

Our screens identified 20 novel strains of arthropod *Rickettsia *from six orders of insects, and these are listed in Table 1. These strains were combined with five previously described arthropod *Rickettsia *(listed at the bottom of Table 1) to give 25 strains in total. We successfully sequenced all four of our chosen genes from 18 of these strains, and one or more genes from the remaining seven.

**Table 1:** *Rickettsia *strains sequenced.

*Rickettsia *obtained from:	Host order	Host species
*This study:*		
Worldwide screen	Lepidoptera	Noctuidae (moth)
	Neuroptera	Chrysopidae (lacewing)
	Coleoptera	Elateridae (beetle)
	Coleoptera	Curculionidae (weevil)
	Diptera	Bombyliidae (bee fly)
	Diptera	Bombyliidae (bee fly)
	Hemiptera	Reduviidae (assassin bug)
	Coleoptera	Meloidae (blister beetle)
	Hemiptera	Cercopidae (spittlebug)
Ladybird screen	Coleoptera	*Subcoccinella vigintiquattuorpunctata *(24 spot ladybird)
	Coleoptera	*Halyzia sedecimguttata *(orange ladybird)
	Coleoptera	*Calvia quattuordecimguttata *(cream spot ladybird)
	Coleoptera	*Coccidula rufa *(ladybird)
	Coleoptera	*Rhizobius chrysomeloides *(ladybird)
	Coleoptera	*Scymnus suturalis *(ladybird)
	Coleoptera	*Adalia bipunctata *(2 spot ladybird) edinburgh
	Coleoptera	*Adalia decempuntata *(10 spot ladybird)
Gall wasp screen	Hymenoptera	*Pediobius rotundatus*
	Hymenoptera	*Aulogymnus balani/skianeuros*
	Hymenoptera	*Aulogymnus trilineatus*
*Previous studies:*		
Jiggins and Tinsley [79]	Coleoptera	*Adalia bipunctata *(2 spot ladybird) moscow
	Coleoptera	*Adalia bipunctata *(2 spot ladybird) cambridge
	Coleoptera	*Adalia bipunctata *(2 spot ladybird) ribe
Chen et al. [14]	Hemiptera	*Acyrthosiphon pisum *(pea aphid)
Lawson et al. [7]	Coleoptera	*Brachys tessellatus *(buprestid beetle)

### Rickettsia phylogeny

To obtain a phylogeny of the genus *Rickettsia*, we combined a concatenated alignment of the four genes we sequenced, with data from other *Rickettsia *strains available from Genbank (accession number available in Additional file 1, Accession numbers of genes used in the phylogenetic analysis). For most of the previously described arthropod *Rickettsia*, only *16S *rDNA sequence is available, and so we allowed for missing data in the alignment where a gene had not been sequenced. Missing data should not decrease phylogenetic resolution for taxa with complete data, and is likely to be a problem for other taxa only when the number of characters is very low [39].

**Figure 1:**
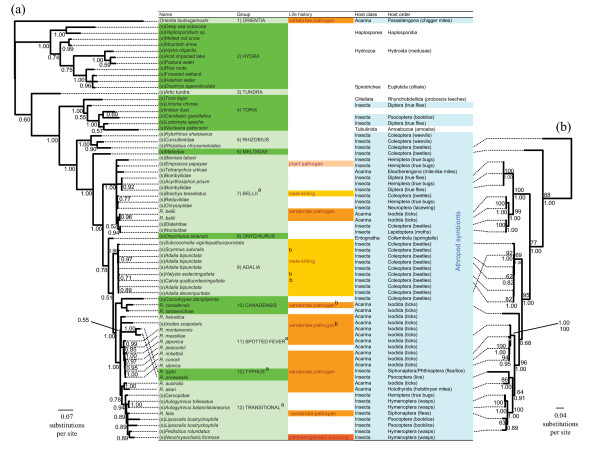
**Phylogeny of *Rickettsia***. The name of the host prefixed by (s) is given where the bacterium does not have a species name, as well as names for each *Rickettsia *group, life history and host order. (a) Bayesian phylogeny using concatenated sequences of *atpA*, *coxA*, *gltA*, *16S*. Posterior support for each node is shown. (b) Maximum likelihood phylogeny based on complete sequences of *atpA*, *coxA *and *gltA*. Bootstrap support is given as a percentage above the node, and posterior support from a Bayesian tree is given as a decimal below the node. ^a^Previously characterised groups. ^b^Only circumstantial evidence connects the trait to the strain.

Figure 1a shows that our concatenated alignment with missing data gave a well-resolved tree with strong support for most nodes. Nevertheless, it is important to determine whether there are conflicting signals between the individual genes. Therefore, we used SH tests to compare our concatenated topology to the maximum likelihood trees inferred from each of the four genes (Table 2). Only the *16S *gene tree topology was marginally significantly different (although this is no longer significant when controlling for multiple tests by Bonferroni correcting the *p-*values).

**Table 2:** Likelihood values of SH tests.

	Likelihood of tree topology			
			
Dataset	Unconstrained	Concatenated	lnL	*p*
*16S*	1486.10	1502.03	31.85	0.045
*AtpA*	2129.98	2140.90	21.85	0.161
*CoxA*	3484.47	3490.98	13.02	0.201
*GltA*	3931.44	3942.56	22.24	0.069

Comparison of the tree topologies obtained from the four genes against the topology of the concatenated dataset using four SH tests. Each dataset was forced to adopt the topology from the concatenated dataset and the log likelihood of this tree was compared with the log likelihood of the unconstrained tree. The taxa used in this analysis are shown in Figure 1b.

It is also important to investigate the influence of missing data on the phylogeny. Therefore, we constructed a second tree that included only taxa with complete sequences for the three genes *atpA*, *coxA *and *gltA *(excluding *16S *due to its marginally significant SH test). This 'complete data' tree is shown in Figure 1b. Overall, the topologies of the two trees are very similar (Figure 1a and 1b), but most nodes had higher support in the tree with complete sequences. In particular, there is strong bootstrap support for the group largely composed of ladybird symbionts in the complete data tree (Figure 1b) but not on the missing data tree (Figure 1a). An exception is the placement of *R. canadensis*, which is uncertain in the complete data tree but is well supported on the missing data tree (probably because the missing data tree includes two closely related taxa; Figure 1a). The composition of the transitional group and the placement of *Rickettsia prowazekii *also differ in the two trees. *Rickettsia *within the typhus group (*R. prowazekii *and *Rickettsia typhi*) are striking in that they reside on longer branches than other *Rickettsia *in the trees. This is indicative of rate heterogeneity, which can cause a long-branch attraction artefact where the taxa will appear in an incorrect place. In the missing data tree the transitional group is monophyletic, while in the complete data tree *R. prowazekii *groups with *Rickettsia akari *(Figure 1a and 1b). However, constraining *R. akari *and the transitional group to be monophyletic in the complete data tree only causes a marginally significant drop in the likelihood (SH test; lnL = 20.003 *p *= 0.066).

**Figure 2:**
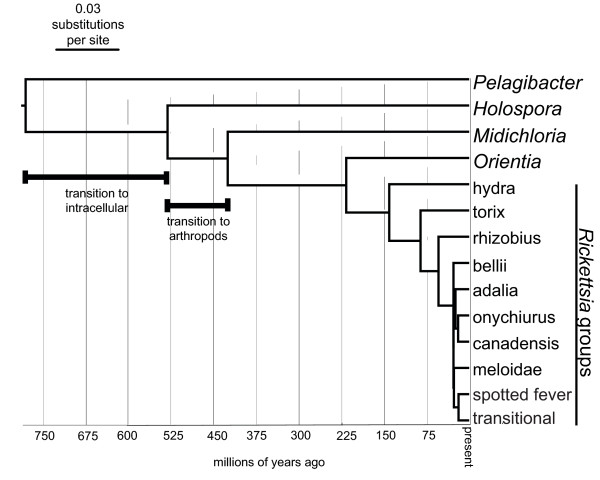
**Relationships and approximate dates of divergence of the major clades within the order Rickettsiales**. The *16S *rDNA phylogeny was reconstructed using one member of each of the groups shown with a molecular clock enforced (enforcing the clock did not reduce the likelihood of the tree: likelihood ratio test lnL = 13.84, df = 12 *p *= 0.311).

Together, these phylogenetic analyses reveal five distinct and well-supported major clades of *Rickettsia *(Figure 1), one (designated the hydra group) containing protist-associated *Rickettsia *and a number with unknown host associations from sequences amplified from environmental samples, a second clade (torix) containing *Rickettsia *from amoeba, leeches and arthropods, a third (rhizobius) contains three beetle *Rickettsia*, a fourth (melloidae) containing a single beetle *Rickettsia*, a fifth (bellii) containing 11 strains of arthropod *Rickettsia *and a sixth clade of diverse bacteria containing both arthropod and vertebrate *Rickettsia*. This final clade can be further subdivided into the following groups: onychiurus, adalia, canadensis, spotted fever group, typhus group and transitional group, although bootstrap support for some of these groupings is less strong (all groups are also summarized in Figure 2).

### Host shifts

By mapping host species onto our phylogeny, we are able to make inferences about patterns of host-switching in the genus. It is clear from Figure 1 that *Rickettsia *bacteria have an extremely diverse host range, occurring in arthropods, vertebrates, plants, amoebas, ciliates, annelids and hydrozoa, and that there have been numerous shifts between these hosts. The earliest shift splits the genus into two major divisions: the hydra and torix groups and all other arthropod *Rickettsia*. As mentioned, the hydra group are symbionts of protists and undetermined hosts. Although one member of this group was found in the marine ciliate *Diophrys *from brackish water [20], and another from a deep sea octocoral, all others are from freshwater environments or damp terrestrial environments. In general it appears that marine *Rickettsia *are rare. Indeed, from over 13 billion open reading frames compiled from marine metagenomic datasets [40] we detected no homologues of greater than 91% identity to the *16S *gene of hydra group *Rickettsia*. The next split in the tree separates all the remaining *Rickettsia *from the torix group (Figure 1) which contains symbionts of leeches (phylum Annelida), an amoeba [36] and arthropods (a sandfly, a cranefly, a biting midge and a booklouse). In the torix group, as with the hydra group, the vast majority of the hosts are aquatic at some stage in their life cycle (the sole exception being the booklouse).

The remainder of the arthropod *Rickettsia*, including all strains sequenced in the present study, form a monophyletic group (Figure 1). Parsimony suggests that the ancestral state of this clade is to infect arthropods, with one or more lineages subsequently evolving to also infect vertebrates. In addition, there have been multiple transitions between blood feeding and non blood feeding insects. Perlman et al. [36] demonstrated that forcing *R. bellii *to group with other blood feeders gives a significantly worse tree. SH tests of our phylogeny showed that forcing *R. canadensis *and *Rickettsia felis *to group with other blood feeders similarly gives a significantly worse fit (SH tests on all groups: *p *< 0.001).

Our results therefore show clearly that there have been numerous host shifts, sometimes between taxonomically distant hosts. However, it is equally clear that related *Rickettsia *tend to share related hosts. Multiple different strains were detected within ladybird beetles, ticks, lice, parasitic wasps and bee-flies, and in all cases, two or more of these strains cluster together. Nevertheless, this pattern does not seem to be explained by ancestral infection followed by co-speciation of parasite and host. From Figure 1a, the three different strains of *Rickettsia *found in *Adalia bipunctata *do not appear to be monophyletic as one of the *A. bipunctata *strains groups with *Adalia decempunctata *with high posterior support. Unfortunately only four parasitoid individuals from the oak gall wasp screen were infected, not allowing us to test the influence of host relatedness, host interaction frequency and geographic isolation on frequency of horizontal transfer events.

In addition to clustering according to host type, Figure 1 also demonstrates phylogenetic clustering by ecology (although it is often difficult to separate these effects). For example, the two major groups of vertebrate *Rickettsia*, the spotted fever or typhus groups, consist solely of vertebrate *Rickettsia*, containing no arthropod *Rickettsia*. However, the transitional group differs from this pattern containing both vertebrate *Rickettsia *and *Rickettsia *infecting non-blood feeding arthropods (Figure 1). A second ecological adaptation to increase transmission is to skew the sex ratio of the host towards females, which are the sex that most efficiently transmits the infection to offspring for vertically transmitted *Rickettsia*. Some of these *Rickettsia *are known or suspected to kill male hosts early in their development, and there appears to be two separate origins of this adaptation on the tree (once within a buprestid beetle in the bellii group and once within ladybirds in the adalia group). There are 11 strains of *Rickettsia *that infect ladybird beetles, and nine of these cluster in a single monophyletic group. The ones that cluster elsewhere are probably not male-killers (male ladybird beetles are also infected at high prevalence [6]). A third possible source of ecological clustering relates to herbivorous hosts. Such clustering may reflect ecology in two possible ways. Firstly, many symbionts are known to supplement their hosts with amino acids that are rare in phloem sap (although a mutualistic role for *Rickettsia *has never been demonstrated). Secondly, *Rickettsia *may be transmitted horizontally through plants (one case is already known). It has previously been asserted that the bellii group consists mainly of herbivorous arthropod symbionts [36]. Four *Rickettsia *in this group are indeed known to infect sap sucking arthropods (a whitefly, a leaf hopper, an aphid and a red spider mite), and three of these group separately from the other members of the bellii group (Figure 1). However, we have uncovered a large number of predatory insect hosts in this group, and sap sucking insects in other groups (a spittlebug symbiont is in the transitional group). Therefore, the view that members of the bellii group are mainly associated with herbivorous arthropods is not supported by these new data. However, it is possible that the DNA signal could have come from the guts of these insects, as abdomens were sometimes extracted where there was not enough ovary tissue (although the signal would not be expected to be strong).

### Recombination

**Figure 3:**
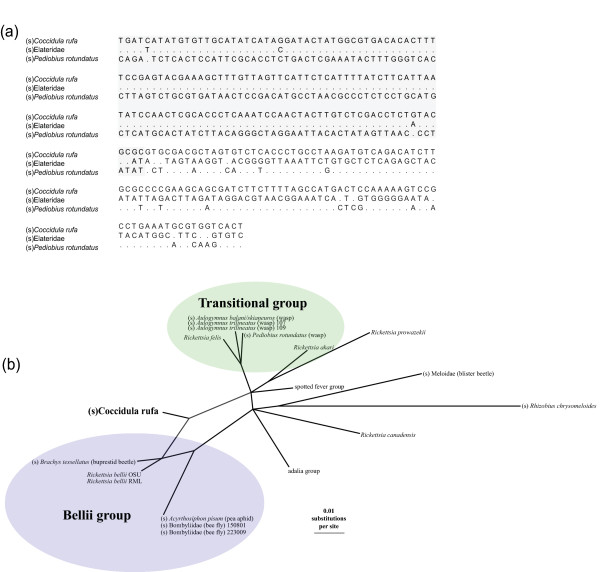
**Sequence alignment and hybridisation network showing the symbiont of *Coccidula rufa *to be a recombinant**. (a) Alignment of concatenated genes *atpA*, *coxA*, *gltA*, *16S *showing just polymorphic sites. Nucleotides that are identical to the *C. rufa *sequence are shown as a dot. The (s)*C. rufa *sequence of *atpA *and *coxA *(shaded) are most similar to (s)Elateridae in the bellii group, while the *gltA *and *16S *sequences (unshaded) are most similar to (s)*Pediobius rotundus *in the transitional group. (b) A hybridisation network of the concatenated sequences of *atpA*, *coxA*, *gltA *and *16S*. A neighbour-net split network was generated and splits were then filtered by weight to include only the (*s*)*C. rufa *split. A hybridisation network was then performed on the split network to provide an explicit example of descent from the two different groups.

Recombination events complicate the inference of species trees, and so it is important to investigate the extent of recombination in the *Rickettsia *genus. We found one clear instance of recent recombination between different *Rickettsia *groups (this taxon was excluded from the analyses above). In the phylogenetic trees of the four individual genes (Additional file 2, Phylogenetic trees of each of the individual genes used in the study), the symbiont of the ladybird *Coccidula rufa *(s*C. rufa*) appears in the transitional group on the *16S *and *gltA *trees, and in the bellii group on the *atpA *and *coxA *trees. An alignment of the polymorphic sites and a hybridisation network indicates that s*C. rufa *is a chimera of sequences from these two groups (Figure 3). To verify that the recombination pattern for s*C. rufa *was not the result of contamination, this result was confirmed by sequencing three strains from different individuals of *C. rufa*. This appears to be the only case of recombination between the four genes because when s*C. rufa *is excluded from analyses, there is little evidence of topological differences between the datasets (see SH tests above).

We did, however, detect some evidence of recombination events within two of the four genes. The maximum χ^2 ^test and phi test identified multiple recombination breakpoints in the *gltA *and *coxA *genes. In *coxA*, the breakpoint pattern indicted that there had been some recombination between an ancestor of the adalia group and of the rhizobius group (maximum χ^2 ^test χ^2 ^= 42.79 *p *< 0.001; phi test *p *< 0.001). In *gltA*, there was evidence of recombination between *R. akari *of the transitional group and the adalia group (maximum χ^2 ^test χ^2 ^= 46.78 *p *< 0.001; phi test *p *= 0.021). In contrast, no recombination was detected within the *16S *and *atpA *genes (maximum χ^2 ^test χ^2 ^= 8.92 *p *= 0.783; phi test *p *= 0.960; maximum χ^2 ^test χ^2 ^= 12.13 *p *= 0.57; phi test *p *= 0.759 (respectively)).

Split networks were constructed for each of the four genes to identify possible sources of conflicting signal and recombination in the data (Additional file 3, Split networks for each of the individual genes used in the study). This method has an advantage over tree-based methods as posterior support and bootstrap values measure robustness solely with respect to sampling error (as opposed to systematic bias), and with large sample size robustness will generally be high as noise in the data is filtered out. The split network constructed for the *16S *gene was tree-like (containing no significant splits). In contrast the other three genes showed a small amount of phylogenetic conflict, with statistical support for two different trees. In all cases, one of these trees corresponded to that shown in Figure 1, suggesting that this tree accurately reflects the evolutionary history of most of the genome. The discrepancies were as follows. The *atpA *split network showed additional support for a tree where *R. prowazekii *is basal to the other vertebrate groups. This pattern is consistent with a tree based on protein alignments of the ten *Rickettsia *genomes [41]. The *coxA *split network supported a closer relationship between *Rickettsia chrysomeloides *symbiont and the adalia group, which is consistent with the recombination pattern for this gene. The *gltA *split network also supported this same relationship although this was not reflected in the recombination breakpoint pattern.

## Discussion

We have identified a large number of new strains of *Rickettsia*, including several new groups, and shown that arthropod *Rickettsia *are both common and diverse. We have also constructed the largest and most robust phylogenetic analysis of the genus to date. Importantly, we used a multiple locus approach, as using single genes to build species phylogenies can seriously confound the true relationship between strains, especially with loci that are prone to recombination [42].

### The evolutionary history of Rickettsia

It is useful to view our results in the context of the evolution of the whole order Rickettsiales. To do this, we have used a molecular clock to date the divergence of different groups, and this is shown in Figure 2. The common ancestor was presumably free-living, as the earliest diverging genus of the order is *Pelagibacter*. *Pelagibacter *species account for 26% of the bacterial rDNA sequences from sea water [43] and have the smallest genomes of free-living bacteria. About 525–775 million years ago there was a transition to living within cells, followed by a split into endosymbionts of protists (*Holospora*) [44,45] and a clade that primarily infects arthropods. *Holospora *species infect the nuclei of paramecium and are generally considered pathogenic to their hosts; for example, *Holospora undulata *can sterilise their hosts, reduce the rate of asexual division and increase host mortality [46]. The most parsimonious interpretation of the tree, therefore, is that the transition to infecting arthropods occurred approximately 425–525 million years ago in this lineage (Figure 2), which can be compared to the first appearance of most metazoan phyla in the Cambrian explosion (approximately 540 million years ago).

All other genera in the order Rickettsiales are associated with arthropods although many have other diverse hosts. The genus *Midichloria *has only been found in Ixodidae ticks, and resides inside mitochondria. Bacteria in the genus *Neorickettsia *are primarily associated with helminths, where they can be transmitted to vertebrates [47]. *Wolbachia *have been described in only arthropods and nematodes, and most are thought to be vertically transmitted (reviewed in [48]). *Ehrlichia *and *Anaplasma *are horizontally transmitted in arthropods and vertebrates [49,50] and *Orientia *are vertically transmitted in mites and can be horizontally transferred to humans [51,52].

The genus *Rickettsia *is approximately 150 million years old (Figure 2). Parsimony would suggest that the common ancestor of *Rickettsia *infected arthropods, and that species in the hydra and torix groups then switched to infect other eukaryotes such as protists, leeches and numerous unidentified hosts (many of which may be protists) (Figures 1 and 3). However, care should be taken with this interpretation, as symbionts of arthropods are more thoroughly sampled than those of other animals. In addition, two patterns call into question the interpretation that the ancestral state was arthropod infection. First, the genome sequence of *R. bellii *includes many genes that are more related to other amoeba symbionts than to other *Rickettsia *[53]. This is compatible with an ancestor of *R. bellii *infecting amoebas and exchanging genes with other amoebal symbionts. Second, of the arthropod hosts within the torix group (three Diptera and a booklouse), all of the Dipteran hosts have larval stages that feed on aquatic microbiota, with the other hosts within the group also being aquatic. Although host switching could occur in either direction, transmission from protist to arthropod is more intuitive given that the related genus *Neorickettsia *is transmitted between hosts through ingestion [47]. Further sampling of other eukaryotic hosts may resolve the question of the ancestral state.

Regardless of this, we have shown that the remaining clade of *Rickettsia *(i.e. those not in the hydra or torix groups) all have associations with arthropods; either as the only known host or in conjunction with a vertebrate or plant host (Figure 1). The rhizobius and meloidae groups, which all infect beetles, diverged from the other taxa early in the evolution of this clade. There was then a rapid radiation about 50 million years ago that led to most of the strains we know of. This includes the bellii group, which is probably the largest group of arthropod *Rickettsia *as it contains all but three strains from the worldwide sample. This sample includes both a diverse array of arthropods (it rarely includes the same host genus twice), and it will tend to pick up high prevalence infections (only a single specimen of each host species was tested).

Our results show clearly that switching between arthropod hosts has been a common feature of *Rickettsia *evolution. Within the genus, closely related bacteria sometimes infect different host phyla and classes (Figure 1), but the genus arose long after the major arthropod orders diverged [54] (Figure 2). However, the host phylogeny is not entirely unrelated to the bacterial phylogeny, and there are many cases of related *Rickettsia *strains infecting related hosts. In the case of many mutualistic symbionts, the bacterial phylogeny precisely mirrors the host phylogeny, indicating that the bacteria and host have co-speciated [55]. However, this is not the case in the *Rickettsia*. Even in the adalia group, where a group of related bacteria all infect related hosts, the host and bacterial phylogenies are different. Therefore, *Rickettsia *symbioses are short-lived on an evolutionary scale, which is consistent with most of these infections being parasitic.

Our analysis has also allowed us to reconstruct the changes in the ecology of the genus. *Rickettsia *are almost entirely restricted to terrestrial and freshwater habitats (Figure 1). Within the genus, there have been three major transitions in life history: becoming sex ratio distorters, arthropod vectored vertebrate pathogens and, in one case, an arthropod vectored plant pathogen. Based on current data, infecting plants and parthenogenesis induction in the arthropod host has arisen only once, and male-killing twice. Until the effect of *R. bellii *on vertebrates in the field has been properly defined, we cannot say for sure how many times vertebrate pathogenesis has evolved.

### Recombination

The recent discovery of plasmids in the genus *Rickettsia *opens up the possibility that horizontal gene transfer may be common between strains [56-59]. Furthermore, there have been reports of recombination between *Rickettsia *strains [60,61]. This has important implications for the evolution of *Rickettsia*, as genes can sweep through different genetic backgrounds of bacterial strains, thereby potentially increasing the spread of genes altering bacterial pathogenicity. Recombination can also complicate the inference of relationships between strains, as recombination violates the assumption that a strain has one evolutionary history.

It is clear from our data that these different genes have very similar phylogenetic histories and recombination must therefore be infrequent (although it is possible that the exchange of plasmids may be common). However, we detected one clear-cut case of recombination between different groups of *Rickettsia*. In the symbiont of the ladybird beetle *C. rufa *(Figure 3) the sequences of *atpA *and *coxA *place (s)*C. rufa *within the bellii group, whereas *gltA *and *16S *place it within the transitional group (Additional file 2, Phylogenetic trees of each of the individual genes used in the study). In the *R. felis *genome (from the transitional group), the gene sequences of *atpA *and *coxA *are approximately 670 kb apart. If this represents one recombination event and the genes are syntenic with the *R. felis *genome, it will have included approximately 45% of the genome. The biggest known recombination event in *Rickettsia*, which occurred in *Rickettsia massiliae*, is a 54 kb segment containing many genes that facilitate conjugal DNA transfer. Intriguingly, although *R. massiliae *is in the spotted fever group, this region of DNA was also thought to originate from the bellii group [58]. As well as this, Gillespie et al. [34] found that many of the genes on the *R. felis *plasmid have a closer relationship to the bellii group. This evidence suggests that conjugation with the bellii group *Rickettsia *may have an important role in the evolution of the groups containing vertebrate pathogens.

We also detected recombination within the *coxA *and *gltA *genes. This is particularly surprising given that the individual gene topologies did not seem to conflict in any way (Table 2). This can only be explained if the recombination event is ancient, and indeed the breakpoint patterns affected all members in particular groups, suggesting that the events pre-dated the divergence of the different groups. Even though recombination machinery has been detected in *Rickettsia *genomes [62], this is the first evidence that housekeeping genes recombine, and could have implications for the inference of relationships, especially since housekeeping genes (in particular *gltA *in *Rickettsia*) are often used to build phylogenies. Therefore recombination should be investigated more fully, especially when using single genes to build phylogenies. These ancient recombination events involve the adalia group and the rhizobius group, as well as the transitional group. This would seem to indicate that recombination is not unique to the bellii and vertebrate groups, and may be widespread throughout all arthropod *Rickettsia *and possibly the other basal groups. However, the recombination signal is different from the above cases, as it is intragenic and over a small area.

### Transmission and population dynamics

It is clear from our data that *Rickettsia *are common and diverse bacteria. However, the basic biology of most of these strains is entirely unknown and it is therefore unclear how these have spread through populations. As *Rickettsia *are primarily intracellular, they cannot survive for long in the external environment (but see [63] for cell-free persistence of related *Wolbachia*). For this reason, they are most readily maintained either by vertical transmission (mother to offspring) in their arthropod hosts or, in the case of blood-sucking arthropods, by horizontal transmission through an infected vertebrate (one case is also known of transmission through a plant [10]). Because infectious transmission between arthropod hosts is thought to be rare, the general view is that exclusively arthropod *Rickettsia *are maintained within a host species primarily by transovarial transmission, and therefore must enhance the fitness of infected females [64]. Some *Rickettsia *raise infected female fitness in an indirect way by manipulating host reproduction towards infected daughters at the expense of sons, either by killing male offspring as embryos (male-killing) or by inducing parthenogenesis [13,65]. The closely related bacterium *O. tsutsugamushi *also causes a female biased sex ratio in its mite host [66]. Theoretically, arthropod *Rickettsia *could also be maintained by directly providing a fitness benefit to infected females as shown for other bacterial groups [67-71], eg by providing essential nutrients or protection from other infective agents. Although, *Rickettsia *are required for egg production in the booklouse *Liposcelis bostrychophila*, and are therefore obligatory, in most cases where the arthropod relationship has been studied in depth, *Rickettsia *are pathogenic [8,72-74] or have no observable effect [75,76], making a mutualistic role for *Rickettsia *in those hosts unlikely.

For those *Rickettsia *that are vertebrate pathogens but vectored by arthropods, the effects of the bacteria on their arthropod hosts are generally poorly understood [72].*Rickettsia prowazekii *is clearly pathogenic to infected lice, and transmission through humans is essential to the maintenance of the bacteria in arthropod populations. In every other case, human infections are accidental, but transmission through other vertebrates may allow the bacteria to persist in populations. Many of the bacteria that can infect vertebrates are also transmitted vertically by the arthropod host [76]. In these cases, even very occasional horizontal transmission through the vertebrate host can enhance the maintenance of bacteria in arthropod populations.

Our data also have implications for transmission. We have shown that *R. felis *(transitional group),*R. canadensis *(canadensis group) and *R. bellii *(bellii group) are more closely related to *Rickettsia *in non-blood feeding hosts than to those found in other blood feeding hosts. Therefore, are these strains even transmitted horizontally? As far as we are aware, even in cases where the bacteria can infect vertebrates (as is the case with *R. felis*), there has been no recorded instance of transmission back to arthropods (i.e. ectoparasites can not pick up the infection from vertebrates). Therefore, while there are multiple origins of infecting blood-feeding arthropods, the ability to be transmitted from vertebrates back into the arthropod host may have arisen once only, and subsequently been lost in the transitional group after the divergence of *R. akari *and *Rickettsia australis*.

We still do not have a complete understanding of how *Rickettsia *are maintained within host populations or how they move horizontally between host species. A better understanding of these dynamic processes can be achieved by detailed studies of representatives from the different groups described here.

## Conclusion

We have identified 20 new arthropod *Rickettsia *and described the major transitions and life-history strategies throughout the phylogeny. This raises many questions about how these bacteria are maintained and spread throughout populations of arthropods and vertebrates. *Rickettsia *are known to distort the sex ratio of their hosts by male-killing and inducing parthenogenesis, and are also horizontally transmitted through vertebrates and plants. However, these phenotypes are probably not manifest in the majority of strains discovered and so there may be other ways in which *Rickettsia *are maintained in host populations. For example, there seem to be intriguing links to host oogenesis in some strains and a possible case of a beneficial effect in the torix group [77]. Exploring the biology of these new strains is essential if we are to learn more about the genus.

## Methods

### Bacterial strains

We obtained most of the *Rickettsia *strains we sequenced from three PCR screens of insects collected in the wild (Table 1). These used primers that amplify the *16S *rDNA of *Rickettsia *[15]. The first screen tested 2149 ladybirds from 21 different species collected from the UK, Germany, Spain and New Zealand for the presence of *Rickettsia *[6]. We sequenced a *Rickettsia *from a single individual from each of the eight species shown to be infected. The second screen tested 1458 individuals of Hymenoptera associated with galls induced by oak gall wasps (Hymenoptera: Cynipidae, Cynipini; [78]), comprising nine species of oak gall wasp, 26 species of associated chalcid parasitoid, and ten species of oak gall wasp inquiline (Hymenoptera: Cynipidae, Synergini) (A Aebi and G Stone, unpublished data). We sequenced a *Rickettsia *from single individuals from three of the four species that were infected. The third study screened 847 individuals, each of which was a different species of arthropod from the classes Arachnida, Entognatha, Malacostraca and Insecta. The individuals from Arachinida comprised six of the order Araneae and one Holothyrida. The five Entognatha were all Collembola and the individual from Malacostraca was from the order Isopoda. The individuals from the Insecta comprised 240 of the order Hymenoptera, 218 Diptera, 206 Coleoptera, 86 Hemiptera, 28 Lepidoptera, nine Orthoptera, nine Thysanoptera, eight Odonata, eight Heteroptera, five Homoptera, five Blattodea, four Neuroptera, three Dermaptera, and one individual each of Mantodea, Pscoptera, Siphonaptera, Strepsiptera, and Trichoptera (L Weinert and J Werren, unpublished data). The insects were collected from worldwide locations. All nine *Rickettsia *isolates from this screen were sequenced. More detailed information on infected and uninfected species from unpublished data can be found in the supplementary information (Additional file 4, The distribution of *Rickettsia *among arthropods). We also included a *Rickettsia *from the pea aphid *Acyrthosiphon pisum *[8], a male-killing *Rickettsia *from the buprestid beetle *Brachys tessellatus *[7] and three *Rickettsia *strains from the ladybird beetle *A. bipunctata*, each of which has been shown to be genetically distinct [73,79].

### PCR and sequencing

To obtain estimates of phylogeny from different portions of the genome, we sequenced four different genes, which are at least 200 kbps apart in the *R. bellii *genome. Of the genes used in a previous study to produce a multi-gene vertebrate *Rickettsia *phylogeny [33], we sequenced *16S *rDNA and *atpA*, which are the only ones that have homologues conserved enough to produce alignments in *O. tsutsugamushi*. We also targeted the *coxA *gene as it is used in *Wolbachia *multilocus strain type analysis [80] and is found in *Orientia *and all *Rickettsia *genomes except for *R. typhi*. We also used the *gltA *gene, which is commonly sequenced from *Rickettsia *strains [30] and, although it is absent from the *O. tsutsugamushi *genome, it is conserved throughout all other Rickettsiales [38]. This provides four genes for our multi-gene analysis. The primers used to amplify the four different genes are given in Table 3[81].

**Table 3:** Primers used for PCR amplification and sequencing.

Gene	Description	Primer name	Primer sequence (5'-3')	Reference
*16S*	*16S *rDNA	27f	AGAGTTTGATCCTGGCTCAG	[81]
		rssur	GAAAGCATCTCTGCGATCCG	[15]
*atpA*	ATP synthase F1 alpha subunit	atpAf2	ATCAAGCGTTGCACAGATAG	this study
		Vitr	CGACTTACCGAAATACCGAC	[33]
		atpA536r	GGAAGTGCCGTAAGTGAACC	this study
*gltA*	citrate synthase	rcit133f	GGTTTTATGTCTACTGCTTCKTG	[10]
		rcit1197r	CATTTCTTTCCATTGTGCCATC	[10]
*coxA*	subunit I of cytochrome C oxidase	coxAf2	ACAGCCGTTGATATGGCTA	this study
		coxA1413r	CATATTCCAACCGGCAAAAG	this study
		coxA322f	GGTGCTCCTGATATGGCATT	this study
		coxAr1	CATATTCCAGCCGGCAAAAG	this study

For *AtpA*, the primers used in tandem are atpAf2 as a forward primer and either VitR or atpA536r as reverse primers. For amplification of *coxA*, the primers used are coxAF2 with coxA1413r or coxA322f with coxAr1.

The PCR products were incubated at 37°C for 40 minutes with shrimp alkaline phosphatase (Promega, Southampton, UK) to digest unincorporated dNTPs and exonuclease I (NEB, Hertfordshire, UK) to digest the PCR primers. They were then sequenced using Big Dye technology (Applied Biosystems, CA) in both directions using the PCR primers and run on a 3730 capillary sequencer (Applied Biosystems, CA).

### Phylogenetic analysis

Nucleotide sequences were edited and assembled using Sequencher 4.1 (GeneCodes, MI), and aligned using the ClustalW application within Bioedit v.7.0.1. All sequences within alignments were checked to ensure they encoded functional proteins (with the exception of the *16S *gene). The model of sequence evolution used for each gene was selected by including only parameters that significantly improved the fit of the model to our data. These parameters were identified by comparing alternative models using hierarchical likelihood ratio tests in the program MODELTEST v.3.7 [82]. The evolutionary models used were as follows: *16S *– HKY+G, *gltA *– K81uf+I+G, *coxA *– GTR+G and *atpA *– GTR+G.

Phylogenetic hypotheses were inferred using maximum likelihood in PAUP v.4.b10 and using the Bayesian MC^3 ^approach implemented in MrBayes v3.1 [83]. We combined our data with published sequences from all the known non-vertebrate *Rickettsia *strains, and all the *Rickettsia *from the ancestral, typhus and transitional groups, as well as *Rickettsia helvetica*, *Rickettsia montanaensis*, *R. massiliae*, *Rickettsia japonica*, *Rickettsia conorii*, *Rickettsia peacockii *and *Rickettsia rickettsii *from the spotted fever group (Figure 1a). We also included *O. tsutsugamushi *as an outgroup (we checked that this species is a genuine outgroup by reconstructing a *16S *rDNA tree rooted with *Wolbachia pipientis*; data not shown). All accession numbers are given in Additional file 1, Accession numbers of genes used in the phylogenetic analysis. Maximum parsimony trees were created using the tree-bisection reconnection branch swapping method, and these were then used both to estimate model parameters and as a starting tree for the maximum likelihood analysis. The maximum likelihood trees were then found using the nearest-neighbour-interchanges branch swapping method. The robustness of the tree topologies was assessed by repeating the analysis using 1000 bootstrapped datasets. The GTR+I+G model of evolution was used for the concatenated dataset of the three genes.

The Bayesian analysis incorporated four Markov chains (three heated and one cold chain), consisting of 1,000,000 generations with sampling every 100 generations. Two simultaneous runs with different random start trees were performed, and the first 25% of samples were discarded as burn-in. For the Bayesian analysis including missing data, the data were partitioned for the four different genes and assigned the appropriate evolutionary model (given above), then unlinked so that the parameters were estimated separately and allowed to have a different evolutionary rate. The MCMC analysis was then run for 6,000,000 generations, after which the standard deviation of split frequencies (a measure of the similarity of the two independent trees in the run) fell below a proposed threshold for model convergence of 0.01 [83]. For the phylogeny that contains missing data, we used only the Bayesian approach.

Split networks for each of the four genes were constructed using the neighbour-net method in SplitsTree4 [84,85]. Networks represent multiple trees simultaneously, and they can therefore identify conflicting signals in the data. These may arise from either genetic exchange between bacterial strains, or from systematic error in the underlying model of evolution. The neighbour-net method computes a matrix of distances (much like the neighbour joining method) and produces a network with weights assigned to each split that are proportional to the number of sites that support the split. We used non-parametric bootstrapping to identify splits supported with > 95% confidence, and only included these statistically significant splits in our network (otherwise representing the data as a bifurcating tree) [85].

### Phylogenetic tests

We tested whether there were significant topological differences between the maximum likelihood trees of the four genes and a tree produced from the concatenated sequences of all four genes using the Shimodaira-Hasegawa test [86]. The test statistic for a given gene is generated by comparing the maximised likelihood score for that gene with topology unconstrained, with the likelihood obtained when topology was fixed at the maximum likelihood topology obtained from the concatenated dataset. The null distribution of the test statistic for a gene is generated from 1000 nonparametric bootstrapped datasets, although to reduce the computational burden, nuisance parameters were fixed at values estimated from the original dataset (RELL method). This test was applied to each of the genes with the *Rickettsia *strain from *C*. *rufa *removed for reason of recombination (see Results).

We tested for recombination between *Rickettsia *strains in two ways. First, we used the maximum χ^2 ^test [87] implemented in RDP v3b22 [88]. This test takes all possible triplets of sequences, removes any gaps, and makes an alignment of just the polymorphic sites. A window is then slid along this alignment in single nucleotide steps. At each position a χ^2 ^statistic is calculated as a measure of the likelihood that recombination has occurred between these sequences. The size of the window was set at approximately 3/4 the numbers of polymorphic sites present for each triplet. To correct for the large number of multiple tests performed, we obtained an analysis-wide significance threshold of χ^2 ^by repeating the analysis on 1000 datasets that were simulated without recombination (simulations performed using Seq-Gen [89]). The maximum χ^2 ^test of recombination is one of the most powerful tests of recombination [90] but it can occasionally falsely infer the presence of recombination under some conditions, such as in regions that contain mutational hot-spots [91]. Therefore we also used the pairwise homoplasy index (PHI) test of recombination [91] implemented in SplitsTree4. The test exploits the fact that when recombination has occurred, sites that are physically close in the sequence should yield compatible phylogenies more often than distant sites. The phi statistic (Φ_*w*_) quantifies the degree of congruence between parsimonious trees at closely-linked sites up to 100 bp (*w *= 100). A *p*-value can then be obtained by comparing this statistic with a distribution of values obtained when the position of sites along the sequence is determined at random. To speed computation, this null distribution can be approximated by a normal distribution, whose mean and variance are calculated analytically from the data.

To date key transitions in the order Rickettsiales, we calibrated a *16S *rDNA phylogeny of the order using the substitution rate of this gene estimated for the endosymbiont *Buchnera *[55]. This tree was reconstructed with a molecular clock enforced. We checked that enforcing a clock did not significantly reduce the likelihood of the tree by comparing the likelihoods of a tree with and without a clock enforced using a likelihood ratio test.

## Authors' contributions

LAW and FMJ conceived the study. LAW, JHW, AA, GNS and FMJ designed the study. LAW and AA collected the data. JHW, GNS and FMJ provided reagents and equipment. LAW and FMJ analysed the data. LAW, JHW, AA, GNS and FMJ interpreted the data. LAW and FMJ drafted the manuscript and JHW, AA and GNS commented on the draft.

## Supplementary Material

Additional file 1 **Table S1**. Accession numbers of genes used in the phylogenetic analysis. Click here for file

Additional file 2 **Figure S1 Phylogenetic trees of each of the individual genes used in the study.** Posterior probabilities are given above the node and maximum likelihood values are given below. Branch lengths are indicated by the scale bar of substitutions per site at the bottom left of each gene tree. Click here for file

Additional file 3 **Figure S2 Split networks for each of the individual genes used in the study.** A test of tree-likeness was carried out on each of the individual gene and only the 95% confidence network is shown, indicating only the statistically significant splits. Branch lengths are indicated by the scale bar of substitutions per site at the bottom left of each gene tree. Click here for file

Additional file 4 **Table S2.** The distribution of *Rickettsia *among arthropods. Incidence data is given for the unpublished wasp and worldwide screen. Click here for file
