# Supplementary material for: Evolution and diversity of Rickettsia bacteria
Source: BMC Biol. 2009 Feb 2;7:6. doi: 10.1186/1741-7007-7-6 (PMC2662801; doi:10.1186/1741-7007-7-6)
Supplement: Additional file 1 — Table S1. Accession numbers of genes used in the phylogenetic analysis. [file 1741-7007-7-6-S1.doc]

| Strain name | *16S* gene | *gltA* gene | *AtpA* gene | *CoxA* gene |
| --- | --- | --- | --- | --- |
| *Orientia tsutsugamushi* | AM494475 | - | AM494477 | AM494478 |
| (s)Deep sea octacoral | DQ395479 | - | - | - |
| (s)*Haplosporidium sp.* | AJ319724 | - | - | - |
| (s)Melted red snow | AJ867656 | - | - | - |
| (s)Mountain snow | AJ867656 | - | - | - |
| (s)*Hydra oligactis* | EF667896 | - | - | - |
| (s)Acid impacted lake | EF520410 | - | - | - |
| (s)Pasture water | EF074039 | - | - | - |
| (s)Rice roots | AM159487 | - | - | - |
| (s)Forested wetland | AF523878 | - | - | - |
| (s)Kalahari water | DQ223223 | - | - | - |
| (s)*Diophrys appendiculata* | AJ630204 | - | - | - |
| (s)Artic tundra | AM945518 | - | - | - |
| (s)*Torix tagoi* | AB066351 | - | - | - |
| (s)*Limonia chorea* | AF322443 | - | - | - |
| (s)Indoor dust | AM697554 | - | - | - |
| (s)*Cerobasis guestfalica* | DQ652596 | - | - | - |
| (s)*Lutzomyia apache* | EU223247 | - | - | - |
| (s)*Nuclearia pattersoni* | AY364636 | - | - | - |
| (s)*Kytorhinus sharpianus* | AB021128 | - | - | - |
| (s)Curculionidae | FJ609387 | - | - | FJ666773 |
| (s)*Rhizobius chrysomeloides* | FJ609388 | FJ666753 | FJ666796 | FJ666774 |
| (s)Meloidae | FJ609389 | FJ666754 | FJ666797 | FJ666775 |
| (s)*Bemisia tabaci* | DQ077707 | DQ077708 | - | - |
| (s)*Empoasca papayae* | U76910 | U76908 | - | - |
| (s)*Tetranychus urticae* | AY753175 | - | - | - |
| (s)Bombyliidae | FJ609390 | FJ666755 | FJ666798 | FJ666776 |
| (s)*Acyrthosiphon pisum* | FJ609391 | FJ666756 | FJ666799 | FJ666777 |
| (s)Bombyliidae | FJ609392 | FJ666757 | FJ666800 | FJ666778 |
| (s)*Brachys tessellatus* | FJ609393 | FJ666758 | FJ666801 | - |
| (s)Reduviidae | FJ609394 | - | - | FJ666779 |
| (s)Chrysopidae | FJ609395 | FJ666759 | - | FJ666780 |
| *R. bellii* | CP000849 | CP000849 | CP000849 | CP000849 |
| *R. bellii* | CP000087 | CP000087 | CP000087 | CP000087 |
| (s)Elateridae | FJ609396 | FJ666760 | FJ666802 | FJ666781 |
| (s)Noctuidae | FJ609397 | FJ666761 | FJ666803 | FJ666782 |
| (s)*Onychiurus sinensis* | AY712949 | - | - | - |
| (s)*Subcoccinella vigintiquattuorpunctata* | FJ609398 | FJ666762 | FJ666804 | FJ666783 |
| (s)*Scymnus suturalis* | FJ609399 | - | FJ666805 | FJ666784 |
| (s)*Adalia bipunctata* (Moscow) | FJ609400 | FJ666765 | FJ666807 | FJ666787 |
| (s)*Adalia bipunctata* (Cambridge) | FJ609401 | FJ666764 | FJ666808 | FJ666786 |
| (s)*Adalia bipunctata* (Ribe) | - | FJ666763 | - | - |
| (s)*Halyzia sedecimguttata* | FJ609402 | FJ666766 | FJ666809 | FJ666788 |
| (s)*Calvia quattuordecimguttata* | FJ609403 | FJ666767 | FJ666810 | FJ666789 |
| (s)*Adalia bipunctata* (Edinburgh) | - | - | FJ666806 | FJ666785 |
| (s)*Adalia decempuntata* | FJ609404 | FJ666768 | FJ666811 | FJ666790 |
| (s)*Coccotrypes dactyliperda* | AY961085 | - | - | - |
| *R. canadensis* | CP000409 | CP000409 | CP000409 | CP000409 |
| *R. tarasevichiae* | AF503168 | AF503167 | - | - |
| *R. helvetica* | L36212 | U59723 | DQ821790 | - |
| (s)*Ixodes scapularis* | AB001518 | - | - | - |
| *R. montanensis* | L36215 | U74756 | AY124737 | - |
| *R. massiliae* | CP000683 | CP000683 | CP000683 | CP000683 |
| *R. japonica* | L36213 | U59724 | DQ821776 | - |
| *R. peacockii* | DQ062433 | DQ100162 | - | - |
| *R. rickettsii* | CP000848 | CP000848 | CP000848 | CP000848 |
| *R. conorii* | AE008647 | AE008647 | AE008647 | AE008647 |
| *R. sibirica* | AABW00000000 | AABW00000000 | AABW00000000 | AABW00000000 |
| *R. typhi* | AE017199 | AE017199 | AE017199 | - |
| *R. prowazekii* | AJ235272 | AJ235272 | AJ235272 | AJ235272 |
| *R. australis* | U17644 | U59718 | DQ821777 | - |
| *R. akari* | CP000847 | CP000847 | CP000847 | CP000847 |
| (s)Cercopidae | - | - | - | FJ666791 |
| (s)*Aulogymnus trilineatus* | FJ609405 | FJ666769 | FJ666812 | FJ666792 |
| (s)*Aulogymnus balani/skianeuros* | FJ609406 | FJ666770 | FJ666813 | FJ666793 |
| *R. felis* | CP000053 | CP000054 | CP000055 | CP000056 |
| (s)*Liposcelis bostrychophila* | DQ407743 | - | - | - |
| (s)*Liposcelis bostrychophila* | DQ652592 | - | - | - |
| (s)*Pediobius rotundatus* | FJ609407 | FJ666771 | FJ666814 | FJ666794 |
| (s)*Neochrysocharis formosa* | AB231472 | - | - | - |
| (s)*Coccidula rufa* | FJ609408 | FJ666772 | FJ666815 | FJ666795 |
