# Supplementary figures and images for: Evolution and diversity of Rickettsia bacteria
Source: BMC Biol. 2009 Feb 2;7:6. doi: 10.1186/1741-7007-7-6 (PMC2662801; doi:10.1186/1741-7007-7-6)

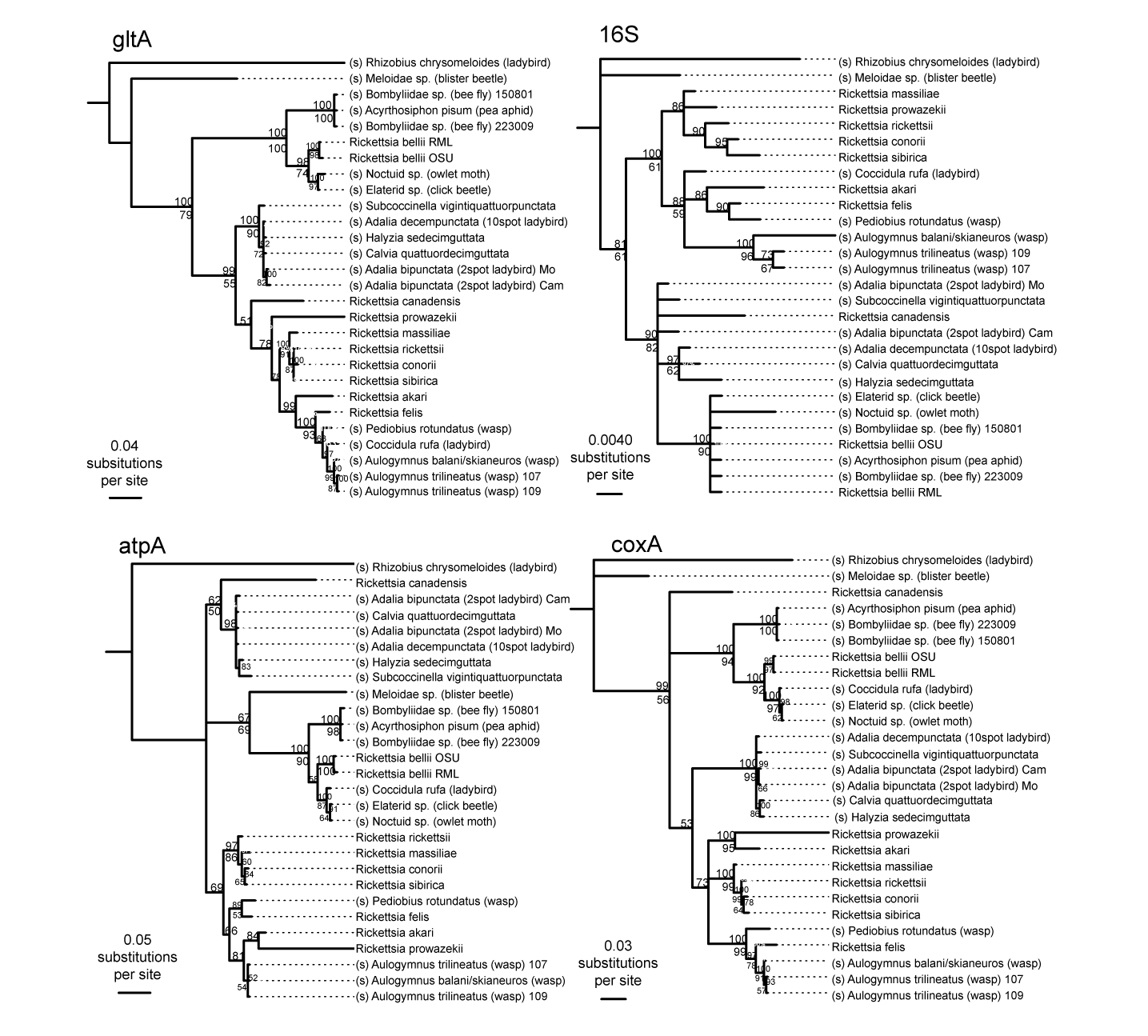

Supplement: Additional file 2 — Figure S1 Phylogenetic trees of each of the individual genes used in the study. Posterior probabilities are given above the node and maximum likelihood values are given below. Branch lengths are indicated by the scale bar of substitutions per site at the bottom left of each gene tree. [file 1741-7007-7-6-S2.jpeg]

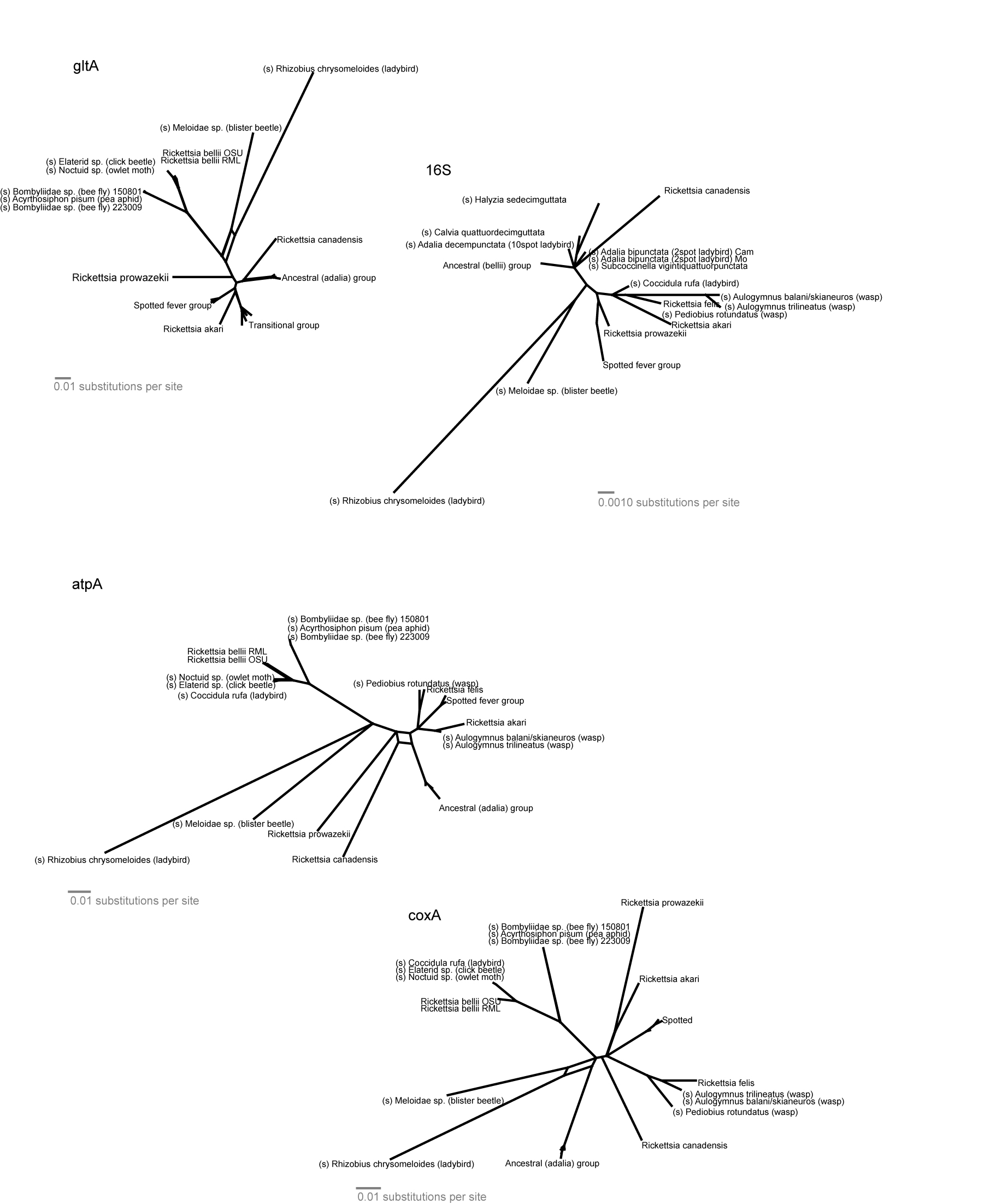

Supplement: Additional file 3 — Figure S2 Split networks for each of the individual genes used in the study. A test of tree-likeness was carried out on each of the individual gene and only the 95% confidence network is shown, indicating only the statistically significant splits. Branch lengths are indicated by the scale bar of substitutions per site at the bottom left of each gene tree. [file 1741-7007-7-6-S3.jpeg]
