# Supplementary material for: Evolution and diversity of Rickettsia bacteria
Source: BMC Biol. 2009 Feb 2;7:6. doi: 10.1186/1741-7007-7-6 (PMC2662801; doi:10.1186/1741-7007-7-6)
Supplement: Additional file 4 — Table S2. The distribution of Rickettsia among arthropods. Incidence data is given for the unpublished wasp and worldwide screen. [file 1741-7007-7-6-S4.doc]

| order | taxon | status | location | n | *R* |
| --- | --- | --- | --- | --- | --- |
| **(WASP SCREEN)** |  |  |  |  |  |
| Hymenoptera | *Amdricus tomentosus* | Gall wasp | Iran | 5 | - |
|  | *Andricus atkinsonae* | Gall wasp | Iran | 3 | - |
|  | *Andricus atkinsonae* | Gall wasp | Iran | 5 | - |
|  | *Andricus ceconni* | Gall wasp | Iran | 20 | - |
|  | *Andricus ceconni* | Gall wasp | Iran | 15 | - |
|  | *Andricus chodjai* | Gall wasp | Iran | 15 | - |
|  | *Andricus curtisii* | Gall wasp | Iran | 15 | - |
|  | *Andricus grossulariae* | Gall wasp | Iran | 40 | - |
|  | *Andricus grossulariae* | Gall wasp | Iran | 10 | - |
|  | *Andricus lucidus* | Gall wasp | Iran | 15 | - |
|  | *Andricus megalucidus* | Gall wasp | Iran | 13 | - |
|  | *Andricus quercustozae* | Gall wasp | Iran | 15 | - |
|  | *Andricus sternlichti* | Gall wasp | Iran | 2 | - |
|  | *Andricus stonei* | Gall wasp | Iran | 15 | - |
|  | *Andricus truncicolus* | Gall wasp | Iran | 5 | - |
|  | *Biorhiza pallida* | Gall wasp | Iran | 9 | - |
|  | *Biorhiza pallida* | Gall wasp | Iran | 1 | - |
|  | *Chilaspis israeli* | Gall wasp | Iran | 15 | - |
|  | *Chilaspis israeli* | Gall wasp | Iran | 10 | - |
|  | *Cynips korsakovi* | Gall wasp | Iran | 8 | - |
|  | *Cynips quercus* | Gall wasp | Iran | 14 | - |
|  | *Cynips quercusfolii* | Gall wasp | Iran | 15 | - |
|  | *Neuroterus saliens ex acorn* | Gall wasp | Iran | 15 | - |
|  | *Neuroterus saliens ex acorn* | Gall wasp | Iran | 15 | - |
|  | *Synergus bechtoldae* | Inquiline | Iran | 16 | - |
|  | *Synergus bechtoldae* | Inquiline | Iran | 5 | - |
|  | *Synergus gallaepomiformis* | Inquiline | Iran | 10 | - |
|  | *Synergus mikoi* | Inquiline | Iran | 17 | - |
|  | *Synergus mikoi* | Inquiline | Iran | 3 | - |
|  | *Synergus pallidipennis* | Inquiline | Iran | 20 | - |
|  | *Synergus pallidipennis* | Inquiline | Iran | 1 | - |
|  | *Synergus umbraculus* | Inquiline | Iran | 25 | - |
|  | *Synergus umbraculus* | Inquiline | Iran | 10 | - |
|  | *Apostrocetus sp nr domenichini* | Parasitoid | Iran | 11 | - |
|  | *Apostrocetus sp nr domenichini* | Parasitoid | Iran | 1 | - |
|  | *Aulogymnus gallarum* | Parasitoid | Iran | 13 | - |
|  | *Aulogymnus gallarum* | Parasitoid | Iran | 4 | - |
|  | *Aulogymnus trilineatus* | Parasitoid | Iran | 15 | - |
|  | *Aulogymnus trilineatus* | Parasitoid | Iran | 7 | - |
|  | *Baryscapus nr anasillus* | Parasitoid | Iran | 2 | - |
|  | *Baryscapus pallidae* | Parasitoid | Iran | 10 | - |
|  | *Baryscapus pallidae* | Parasitoid | Iran | 5 | - |
|  | *Cecidostiba fungosa* | Parasitoid | Iran | 15 | - |
|  | *Cecidostiba fungosa* | Parasitoid | Iran | 6 | - |
|  | *Cyrtoptyx robustus* | Parasitoid | Iran | 2 | - |
|  | *Eupelmus annulatus* | Parasitoid | Iran | 7 | - |
|  | *Eupelmus annulatus* | Parasitoid | Iran | 5 | - |
|  | *Eupelmus urozonus* | Parasitoid | Iran | 9 | - |
|  | *Eupelmus urozonus* | Parasitoid | Iran | 5 | - |
|  | *Eurytoma rosae* | Parasitoid | Iran | 1 | - |
|  | *Eurytoma brunniventris* | Parasitoid | Iran | 15 | - |
|  | *Eurytoma brunniventris* | Parasitoid | Iran | 4 | - |
|  | *Eurytoma brunniventris* | Parasitoid | Iran | 5 | - |
|  | *Eurytoma pistacina* | Parasitoid | Iran | 21 | - |
|  | *Eurytoma pistacina* | Parasitoid | Iran | 5 | - |
|  | *Eurytoma rosae* | Parasitoid | Iran | 10 | - |
|  | *Eurytoma rosae* | Parasitoid | Iran | 2 | - |
|  | *Eurytoma sp.* | Parasitoid | Iran | 10 | - |
|  | *Ichneumonidae* | Parasitoid | Iran | 1 | - |
|  | *Megastigmus dorsalis* | Parasitoid | Iran | 9 | - |
|  | *Megastigmus dorsalis* | Parasitoid | Iran | 4 | - |
|  | *Megastigmus dorsalis main clade* | Parasitoid | Iran | 14 | - |
|  | *Megastigmus dorsalis* | Parasitoid | Iran | 4 | - |
|  | *Megastigmus stigmatizans* | Parasitoid | Iran | 3 | - |
|  | *Megastigmus stigmatizans* | Parasitoid | Iran | 4 | - |
|  | *Ormyrus nitidulus* | Parasitoid | Iran | 1 | - |
|  | *Ormyrus nitidulus* | Parasitoid | Iran | 10 | - |
|  | *Ormyrus nitidulus* | Parasitoid | Iran | 4 | - |
|  | *Ormyrus pomaceus* | Parasitoid | Iran | 16 | - |
|  | *Ormyrus pomaceus* | Parasitoid | Iran | 8 | - |
|  | *Pediobius rotundatus* | Parasitoid | Iran | 2 | - |
|  | *Pediobius rotundatus* | Parasitoid | Iran | 5 | + (1/5) |
|  | *Pteromalus bedeguaris* | Parasitoid | Iran | 10 | - |
|  | *Pteromalus bedeguaris* | Parasitoid | Iran | 5 | - |
|  | *Sycophila biguttata* | Parasitoid | Iran | 19 | - |
|  | *Sycophila biguttata* | Parasitoid | Iran | 10 | - |
|  | *Sycophila iracemae* | Parasitoid | Iran | 5 | - |
|  | *Sycophila iracemae* | Parasitoid | Iran | 4 | - |
|  | *Sycophila variegata* | Parasitoid | Iran | 23 | - |
|  | *Sycophila variegata* | Parasitoid | Iran | 12 | - |
|  | *Torymus auratus* | Parasitoid | Iran | 5 | - |
|  | *Torymus geranii* | Parasitoid | Iran | 10 | - |
|  | *Torymus geranii* | Parasitoid | Iran | 10 | - |
|  | *Andricus caputmedusae* | Gall wasp | Hungary | 10 | - |
|  | *Andricus coriarius* | Gall wasp | Hungary | 5 | - |
|  | *Andricus coronatus* | Gall wasp | Hungary | 2 | - |
|  | *Andricus gemmeus* | Gall wasp | Hungary | 1 | - |
|  | *Andricus glutinosus* | Gall wasp | Hungary | 10 | - |
|  | *Andricus glutinosus* | Gall wasp | Hungary | 4 | - |
|  | *Andricus hungaricus* | Gall wasp | Hungary | 10 | - |
|  | *Andricus lucidus* | Gall wasp | Hungary | 14 | - |
|  | *Andricus lucidus* | Gall wasp | Hungary | 14 | - |
|  | *Andricus quercuscalicis* | Gall wasp | Hungary | 13 | - |
|  | *Andricus quercustozae* | Gall wasp | Hungary | 11 | - |
|  | *Andricus quercustozae* | Gall wasp | Hungary | 4 | - |
|  | *Ceroptres cerri* | Inquiline | Hungary | 18 | - |
|  | *Ceroptres clavicornis* | Inquiline | Hungary | 7 | - |
|  | *Saphronecrus hayni* | Inquiline | Hungary | 9 | - |
|  | *Saphronecrus hayni* | Inquiline | Hungary | 2 | - |
|  | *Synergus consobrinus* | Inquiline | Hungary | 8 | - |
|  | *Synergus consobrinus* | Inquiline | Hungary | 7 | - |
|  | *Synergus gallaepomiformis* | Inquiline | Hungary | 15 | - |
|  | *Synergus gallaepomiformis* | Inquiline | Hungary | 9 | - |
|  | *Synergus hayneanus* | Inquiline | Hungary | 18 | - |
|  | *Synergus hayneanus* | Inquiline | Hungary | 6 | - |
|  | *Synergus incrassatus* | Inquiline | Hungary | 9 | - |
|  | *Synergus incrassatus* | Inquiline | Hungary | 9 | - |
|  | *Synergus pallidipennis* | Inquiline | Hungary | 17 | - |
|  | *Synergus pallidipennis* | Inquiline | Hungary | 6 | - |
|  | *Synergus pallipes* | Inquiline | Hungary | 4 | - |
|  | *Synergus pallipes* | Inquiline | Hungary | 4 | - |
|  | *Synergus umbraculus* | Inquiline | Hungary | 9 | - |
|  | *Synergus umbraculus* | Inquiline | Hungary | 3 | - |
|  | *Aulogymnus ballani/skianeuros* | Parasitoid | Hungary | 1 | + |
|  | *Aulogymnus gallarum* | Parasitoid | Hungary | 16 | - |
|  | *Aulogymnus gallarum* | Parasitoid | Hungary | 7 | - |
|  | *Aulogymnus trilineatus* | Parasitoid | Hungary | 15 | + (2/15) |
|  | *Aulogymnus trilineatus* | Parasitoid | Hungary | 5 | - |
|  | *Caenacis lauta* | Parasitoid | Hungary | 6 | - |
|  | *Eurytoma brunniventris* | Parasitoid | Hungary | 4 | - |
|  | *Eurytoma rosae* | Parasitoid | Hungary | 5 | - |
|  | *Megastigmus dorsalis main clade* | Parasitoid | Hungary | 10 | - |
|  | *Megastigmus dorsalis main clade* | Parasitoid | Hungary | 5 | - |
|  | *Megastigmus dorsalis* | Parasitoid | Hungary | 6 | - |
|  | *Megastigmus dorsalis* | Parasitoid | Hungary | 10 | - |
|  | *Megastigmus stigmatizans* | Parasitoid | Hungary | 14 | - |
|  | *Megastigmus stigmatizans* | Parasitoid | Hungary | 4 | - |
|  | *Mesopolobus albitarsus* | Parasitoid | Hungary | 9 | - |
|  | *Mesopolobus albitarsus* | Parasitoid | Hungary | 1 | - |
|  | *Mesopolobus amaenus* | Parasitoid | Hungary | 5 | - |
|  | *Mesopolobus fasciventris* | Parasitoid | Hungary | 5 | - |
|  | *Mesopolobus fasciventris* | Parasitoid | Hungary | 1 | - |
|  | *Mesopolobus fuscipes* | Parasitoid | Hungary | 15 | + (2/15) |
|  | *Mesopolobus fuscipes* | Parasitoid | Hungary | 7 | - |
|  | *Mesopolobus sericeus* | Parasitoid | Hungary | 1 | - |
|  | *Mesopolobus tarsatus* | Parasitoid | Hungary | 2 | - |
|  | *Mesopolobus tibialis* | Parasitoid | Hungary | 15 | - |
|  | *Mesopolobus tibialis* | Parasitoid | Hungary | 5 | - |
|  | *Mesopolobus xanthocesus* | Parasitoid | Hungary | 16 | - |
|  | *Mesopolobus xanthocesus* | Parasitoid | Hungary | 5 | - |
|  | *Ormyrus nitidulus* | Parasitoid | Hungary | 10 | - |
|  | *Ormyrus nitidulus* | Parasitoid | Hungary | 9 | - |
|  | *Ormyrus pomaceus* | Parasitoid | Hungary | 15 | - |
|  | *Ormyrus pomaceus* | Parasitoid | Hungary | 4 | - |
|  | *Sycophila biguttata* | Parasitoid | Hungary | 10 | - |
|  | *Sycophila iracemae* | Parasitoid | Hungary | 9 | - |
|  | *Sycophila variegata* | Parasitoid | Hungary | 7 | - |
|  | *Sycophila variegata* | Parasitoid | Hungary | 5 | - |
|  | *Torymus auratus* | Parasitoid | Hungary | 9 | - |
|  | *Torymus auratus* | Parasitoid | Hungary | 6 | - |
|  | *Torymus erucarum* | Parasitoid | Hungary | 15 | - |
|  | *Torymus erucarum* | Parasitoid | Hungary | 5 | - |
|  | *Torymus flavipes* | Parasitoid | Hungary | 20 | - |
|  | *Torymus flavipes* | Parasitoid | Hungary | 5 | - |
|  | *Torymus geranii* | Parasitoid | Hungary | 8 | - |
|  | *Torymus geranii* | Parasitoid | Hungary | 1 | - |
| **(WORLD- WIDE SCREEN)** |  |  |  |  |  |
| Isopoda | *unknown sp.* | na | Chile | 1 | - |
| Araneae | *unknown sp.* | na | Mexico | 1 | - |
|  | *Clubionidae* | na | Mexico | 1 | - |
|  | *Gnaphosidae sp.1* | na | Mexico | 1 | - |
|  | *Gnaphosidae sp. 2* | na | Mexico | 1 | - |
|  | *Salticidae* | na | Mexico | 1 | - |
|  | *Salticidae* | na | Mexico | 1 | - |
| Holothyrida | *unknown sp.* | na | Mexico | 1 | - |
| Collembola | *unknown sp. 1* | na | Chile | 1 | - |
|  | *unknown sp. 2* | na | Chile | 1 | - |
|  | *unknown sp. 3* | na | Chile | 1 | - |
|  | *unknown sp. 4* | na | Chile | 1 | - |
|  | *unknown sp. 5* | na | South Africa | 1 | - |
| Blattodea | *unknown sp. 1* | na | Ghana | 1 | - |
|  | *unknown sp. 2* | na | Chile | 1 | - |
|  | *unknown sp. 3* | na | Chile | 1 | - |
|  | *Blattidae sp. 1* | na | Mexico | 1 | - |
|  | *Blattidae sp. 2* | na | Mexico | 1 | - |
| Coleoptera | *unknown sp. 1* | na | Mexico | 1 | - |
|  | *unknown sp. 2* | na | Mexico | 1 | - |
|  | *unknown sp. 3* | na | New York, USA | 1 | - |
|  | *unknown sp. 4* | na | Mexico | 1 | - |
|  | *unknown sp. 5* | na | Ghana | 1 | - |
|  | *unknown sp. 6* | na | Ghana | 1 | - |
|  | *unknown sp. 7* | na | Mexico | 1 | - |
|  | *unknown sp. 8* | na | India | 1 | - |
|  | *unknown sp. 9* | na | Ghana | 1 | - |
|  | *unknown sp. 10* | na | Mexico | 1 | - |
|  | *unknown sp. 11* | na | Mexico | 1 | - |
|  | *unknown sp. 12* | na | Mexico | 1 | - |
|  | *unknown sp. 13* | na | Mexico | 1 | - |
|  | *unknown sp. 14* | na | Panama | 1 | - |
|  | *unknown sp. 15* | na | Panama | 1 | - |
|  | *unknown sp. 16* | na | Panama | 1 | - |
|  | *unknown sp. 17* | na | Panama | 1 | - |
|  | *unknown sp. 18* | na | Panama | 1 | - |
|  | *unknown sp. 19* | na | Panama | 1 | - |
|  | *unknown sp. 20* | na | Panama | 1 | - |
|  | *unknown sp. 21* | na | Panama | 1 | - |
|  | *Anthicidae* |  | Mexico |  | - |
|  | *Anthribidae sp. 1* | na | Ghana | 1 | - |
|  | *Anthribidae sp. 2* | na | Mexico | 1 | - |
|  | *Anthribidae sp. 3* | na | Mexico | 1 | - |
|  | *Anthribidae sp. 4* | na | Mexico | 1 | - |
|  | *Brentidae sp. 1* | na | Ghana | 1 | - |
|  | *Brentidae sp. 2* | na | Papua New Guinea | 1 | - |
|  | *Buprestidae sp. 1* | na | Mexico | 1 | - |
|  | *Buprestidae sp. 2* | na | Mexico | 1 | - |
|  | *Buprestidae sp. 3* | na | Ghana | 1 | - |
|  | *Carabidae sp. 1* | na | New York, USA | 1 | - |
|  | *Carabidae sp. 2* | na | New York, USA | 1 | - |
|  | *Carabidae sp. 3* | na | Mexico | 1 | - |
|  | *Carabidae sp. 4* | na | Mexico | 1 | - |
|  | *Carabidae sp. 5* | na | Mexico | 1 | - |
|  | *Carabidae sp. 6* | na | Mexico | 1 | - |
|  | *Carabidae sp. 7* | na | Mexico | 1 | - |
|  | *Carabidae sp. 8* | na | Mexico | 1 | - |
|  | *Carabidae sp. 9* | na | Mexico | 1 | - |
|  | *Carabidae sp. 10* | na | Mexico | 1 | - |
|  | *Carabidae sp. 11* | na | Mexico | 1 | - |
|  | *Carabidae sp. 12* | na | Mexico | 1 | - |
|  | *Carabidae sp. 13* | na | Mexico | 1 | - |
|  | *Carabidae sp. 14* | na | Mexico | 1 | - |
|  | *Carabidae sp. 15* | na | Mexico | 1 | - |
|  | *Carabidae sp. 16* | na | Mexico | 1 | - |
|  | *Carabidae sp. 17* | na | Mexico | 1 | - |
|  | *Carabidae sp. 18* | na | Mexico | 1 | - |
|  | *Carabidae sp. 19* | na | Mexico | 1 | - |
|  | *Carabidae sp. 20* | na | Mexico | 1 | - |
|  | *Carabidae sp. 21* | na | Mexico | 1 | - |
|  | *Carabidae sp. 22* | na | Mexico | 1 | - |
|  | *Carabidae sp. 23* | na | Mexico | 1 | - |
|  | *Carabidae sp. 24* | na | Mexico | 1 | - |
|  | *Carabidae sp. 25* | na | Mexico | 1 | - |
|  | *Carabidae sp. 26* | na | Mexico | 1 | - |
|  | *Carabidae sp. 27* | na | Mexico | 1 | - |
|  | *Carabidae sp. 28* | na | Mexico | 1 | - |
|  | *Carabidae sp. 29* | na | Mexico | 1 | - |
|  | *Carabidae sp. 30* | na | Mexico | 1 | - |
|  | *Carabidae sp. 31* | na | Ghana | 1 | - |
|  | *Carabidae sp. 32* | na | Ghana | 1 | - |
|  | *Carabidae sp. 33* | na | Ghana | 1 | - |
|  | *Carabidae sp. 34* | na | Ghana | 1 | - |
|  | *Carabidae sp. 35* | na | Ghana | 1 | - |
|  | *Carabidae sp. 36* | na | Ghana | 1 | - |
|  | *Carabidae sp. 37* | na | Ghana | 1 | - |
|  | *Carabidae sp. 38* | na | Ghana | 1 | - |
|  | *Carabidae sp. 39* | na | Ghana | 1 | - |
|  | *Carabidae sp. 40* | na | India | 1 | - |
|  | *Carabidae sp. 41* | na | India | 1 | - |
|  | *Carabidae sp. 42* | na | India | 1 | - |
|  | *Cerambycidae sp. 1* | na | Mexico | 1 | - |
|  | *Cerambycidae sp. 2* | na | Ghana | 1 | - |
|  | *Cerambycidae sp. 3* | na | India | 1 | - |
|  | *Cerambycidae sp. 4* | na | Panama | 1 | - |
|  | *Cerambycidae sp. 5* | na | Papua New Guinea | 1 | - |
|  | *Cerambycidae sp. 6* | na | Papua New Guinea | 1 | - |
|  | *Chrysomelidae sp. 1* | na | Mexico | 1 | - |
|  | *Chrysomelidae sp. 2* | na | Mexico | 1 | - |
|  | *Chrysomelidae sp. 3* | na | New York, USA | 1 | - |
|  | *Chrysomelidae sp. 4* | na | Ghana | 1 | - |
|  | *Chrysomelidae sp. 5* | na | India | 1 | - |
|  | *Chrysomelidae sp. 6* | na | South Africa | 1 | - |
|  | *Chrysomelidae sp. 7* | na | South Africa | 1 | - |
|  | *Chrysomelidae sp. 8* | na | South Africa | 1 | - |
|  | *Chrysomelidae sp. 9* | na | South Africa | 1 | - |
|  | *Chrysomelidae sp. 10* | na | South Africa | 1 | - |
|  | *Chrysomelidae sp. 11* | na | South Africa | 1 | - |
|  | *Chrysomelidae sp. 12* | na | South Africa | 1 | - |
|  | *Chrysomelidae sp. 13* | na | South Africa | 1 | - |
|  | *Chrysomelidae sp. 14* | na | South Africa | 1 | - |
|  | *Chrysomelidae sp. 15* | na | Papua New Guinea | 1 | - |
|  | *Chrysomelidae sp. 16* | na | Papua New Guinea | 1 | - |
|  | *Cleridae sp. 1* | na | Mexico | 1 | - |
|  | *Cleridae sp. 2* | na | Ghana | 1 | - |
|  | *Cleridae sp. 3* | na | Papua New Guinea | 1 | - |
|  | *Cleridae sp. 4* | na | Papua New Guinea | 1 | - |
|  | *Coccinellidae sp. 1* | na | New York, USA | 1 | - |
|  | *Coccinellidae sp. 2* | na | Mexico | 1 | - |
|  | *Curculionidae sp. 1* | na | Mexico | 1 | - |
|  | *Curculionidae sp. 2* | na | India | 1 | - |
|  | *Curculionidae sp. 3* | na | India | 1 | - |
|  | *Curculionidae sp. 4* | na | South Africa | 1 | - |
|  | *Curculionidae sp. 5* | na | South Africa | 1 | - |
|  | *Curculionidae sp. 6* | na | Chile | 1 | + |
|  | *Curculionidae sp. 7* | na | Papua New Guinea | 1 | - |
|  | *Curculionidae sp. 8* | na | Papua New Guinea | 1 | - |
|  | *Curculionidae sp. 9* | na | Papua New Guinea | 1 | - |
|  | *Dytiscidae sp. 1* | na | India | 1 | - |
|  | *Dytiscidae sp. 2* | na | India | 1 | - |
|  | *Elateridae sp. 1* | na | Mexico | 1 | - |
|  | *Elateridae sp. 2* | na | Mexico | 1 | - |
|  | *Elateridae sp. 3* | na | Ghana | 1 | - |
|  | *Elateridae sp. 4* | na | Ghana | 1 | - |
|  | *Elateridae sp. 5* | na | Ghana | 1 | - |
|  | *Elateridae sp. 6* | na | Papua New Guinea | 1 | + |
|  | *Endomychidae* | na | Ghana | 1 | - |
|  | *Erotylidae sp. 1* | na | Mexico | 1 | - |
|  | *Erotylidae sp. 2* | na | Ghana | 1 | - |
|  | *Heteroceridae* | na | India | 1 | - |
|  | *Hydrophilidae sp. 1* | na | India | 1 | - |
|  | *Hydrophilidae sp. 2* | na | India | 1 | - |
|  | *Hydrophilidae sp. 3* | na | India | 1 | - |
|  | *Hydrophilidae sp. 4* | na | India | 1 | - |
|  | *Hydrophilidae sp. 5* | na | India | 1 | - |
|  | *Hydrophilidae sp. 6* | na | India | 1 | - |
|  | *Hydrophilidae sp. 7* | na | India | 1 | - |
|  | *Hydrophilidae sp. 8* | na | Ghana | 1 | - |
|  | *Hydrophilidae sp. 9* | na | Ghana | 1 | - |
|  | *Hydrophilidae sp. 10* | na | Mexico | 1 | - |
|  | *Lagriidae sp. 1* | na | Papua New Guinea | 1 | - |
|  | *Lagriidae sp. 2* | na | Papua New Guinea | 1 | - |
|  | *Lampyridae sp. 1* | na | Chile | 1 | - |
|  | *Lampyridae sp. 2* | na | New York, USA | 1 | - |
|  | *Lampyridae sp. 3* | na | Panama | 1 | - |
|  | *Lampyridae sp. 4* | na | Panama | 1 | - |
|  | *Lycidae* | na | Papua New Guinea | 1 | - |
|  | *Meloidae sp. 1* | na | Mexico | 1 | - |
|  | *Meloidae sp. 2* | na | Mexico | 1 | - |
|  | *Meloidae sp. 3* | na | Mexico | 1 | - |
|  | *Meloidae sp. 4* | na | Panama | 1 | + |
|  | *Mordellidae sp. 1* | na | Mexico | 1 | - |
|  | *Mordellidae sp. 2* | na | Ghana | 1 | - |
|  | *Mordellidae sp. 3* | na | Ghana | 1 | - |
|  | *Nitidulidae* | na | Mexico | 1 | - |
|  | *Ochodaeidae* | na | Mexico | 1 | - |
|  | *Passalidae* | na | Panama | 1 | - |
|  | *Scarabaeidae sp. 1* | na | Mexico | 1 | - |
|  | *Scarabaeidae sp. 2* | na | Mexico | 1 | - |
|  | *Scarabaeidae sp. 3* | na | Mexico | 1 | - |
|  | *Scarabaeidae sp. 4* | na | Mexico | 1 | - |
|  | *Scarabaeidae sp. 5* | na | Ghana | 1 | - |
|  | *Scarabaeidae sp. 6* | na | Ghana | 1 | - |
|  | *Scarabaeidae sp. 7* | na | Ghana | 1 | - |
|  | *Scarabaeidae sp. 8* | na | India | 1 | - |
|  | *Scarabaeidae sp. 9* | na | India | 1 | - |
|  | *Scarabaeidae sp. 10* | na | India | 1 | - |
|  | *Scarabaeidae sp. 11* | na | India | 1 | - |
|  | *Scarabaeidae sp. 12* | na | India | 1 | - |
|  | *Scarabaeidae sp. 13* | na | Panama | 1 | - |
|  | *Scarabaeidae sp. 14* | na | Panama | 1 | - |
|  | *Scarabaeidae sp. 15* | na | Panama | 1 | - |
|  | *Scarabaeidae sp. 16* | na | Panama | 1 | - |
|  | *Staphylinidae sp. 1* | na | Mexico | 1 | - |
|  | *Staphylinidae sp. 2* | na | Mexico | 1 | - |
|  | *Staphylinidae sp. 3* | na | Ghana | 1 | - |
|  | *Staphylinidae sp. 4* | na | Ghana | 1 | - |
|  | *Staphylinidae sp. 5* | na | Ghana | 1 | - |
|  | *Staphylinidae sp. 6* | na | Ghana | 1 | - |
|  | *Staphylinidae sp. 7* | na | Ghana | 1 | - |
|  | *Staphylinidae sp. 8* | na | Ghana | 1 | - |
|  | *Staphylinidae sp. 9* | na | Ghana | 1 | - |
|  | *Staphylinidae sp. 10* | na | Ghana | 1 | - |
|  | *Staphylinidae sp. 11* | na | India | 1 | - |
|  | *Staphylinidae sp. 12* | na | India | 1 | - |
|  | *Staphylinidae sp. 13* | na | India | 1 | - |
|  | *Staphylinidae sp. 14* | na | India | 1 | - |
|  | *Staphylinidae sp. 15* | na | India | 1 | - |
|  | *Staphylinidae sp. 16* | na | South Africa | 1 | - |
|  | *Staphylinidae sp. 17* | na | South Africa | 1 | - |
|  | *Staphylinidae sp. 18* | na | South Africa | 1 | - |
|  | *Staphylinidae sp. 19* | na | Chile | 1 | - |
|  | *Staphylinidae sp. 20* | na | Chile | 1 | - |
|  | *Staphylinidae sp. 21* | na | Chile | 1 | - |
|  | *Staphylinidae sp. 22* | na | Panama | 1 | - |
|  | *Tenebrionidae sp. 1* | na | Mexico | 1 | - |
|  | *Tenebrionidae sp. 2* | na | Mexico | 1 | - |
|  | *Tenebrionidae sp. 3* | na | Ghana | 1 | - |
|  | *Tenebrionidae sp. 4* | na | Ghana | 1 | - |
|  | *Tenebrionidae sp. 5* | na | Ghana | 1 | - |
|  | *Tenebrionidae sp. 6* | na | Ghana | 1 | - |
|  | *Tenebrionidae sp. 7* | na | Ghana | 1 | - |
|  | *Tenebrionidae sp. 8* | na | Ghana | 1 | - |
|  | *Tenebrionidae sp. 9* | na | Ghana | 1 | - |
|  | *Tenebrionidae sp. 10* | na | Ghana | 1 | - |
|  | *Tenebrionidae sp. 11* | na | India | 1 | - |
|  | *Trogidae sp. 1* | na | Mexico | 1 | - |
|  | *Trogidae sp. 2* | na | Mexico | 1 | - |
|  | *Trogidae sp. 3* | na | Mexico | 1 | - |
| Dermaptera | *unknown sp. 1* | na | Ghana | 1 | - |
|  | *unknown sp. 2* | na | Chile | 1 | - |
|  | *unknown sp. 3* | na | unknown | 1 | - |
| Diptera | *unknown sp. 1* | na | Ghana | 1 | - |
|  | *unknown sp. 2* | na | Mexico | 1 | - |
|  | *unknown sp. 3* | na | Mexico | 1 | - |
|  | *unknown sp. 4* | na | Mexico | 1 | - |
|  | *unknown sp. 5* | na | Mexico | 1 | - |
|  | *unknown sp. 6* | na | Mexico | 1 | - |
|  | *unknown sp. 7* | na | Mexico | 1 | - |
|  | *unknown sp. 8* | na | Mexico | 1 | - |
|  | *unknown sp. 9* | na | Mexico | 1 | - |
|  | *unknown sp. 10* | na | Mexico | 1 | - |
|  | *unknown sp. 11* | na | Mexico | 1 | - |
|  | *unknown sp. 12* | na | Mexico | 1 | - |
|  | *unknown sp. 13* | na | Mexico | 1 | - |
|  | *unknown sp. 14* | na | Mexico | 1 | - |
|  | *unknown sp. 15* | na | Mexico | 1 | - |
|  | *unknown sp. 16* | na | Mexico | 1 | - |
|  | *unknown sp. 17* | na | Mexico | 1 | - |
|  | *unknown sp. 18* | na | Mexico | 1 | - |
|  | *unknown sp. 19* | na | Mexico | 1 | - |
|  | *unknown sp. 20* | na | Mexico | 1 | - |
|  | *unknown sp. 21* | na | Mexico | 1 | - |
|  | *unknown sp. 22* | na | Mexico | 1 | - |
|  | *unknown sp. 23* | na | Mexico | 1 | - |
|  | *unknown sp. 24* | na | Mexico | 1 | - |
|  | *unknown sp. 25* | na | Mexico | 1 | - |
|  | *unknown sp. 26* | na | Mexico | 1 | - |
|  | *unknown sp. 27* | na | Mexico | 1 | - |
|  | *unknown sp. 28* | na | Mexico | 1 | - |
|  | *unknown sp. 29* | na | Mexico | 1 | - |
|  | *unknown sp. 30* | na | Mexico | 1 | - |
|  | *unknown sp. 31* | na | Mexico | 1 | - |
|  | *unknown sp. 32* | na | Mexico | 1 | - |
|  | *unknown sp. 33* | na | New York, USA | 1 | - |
|  | *unknown sp. 34* | na | New York, USA | 1 | - |
|  | *unknown sp. 35* | na | New York, USA | 1 | - |
|  | *unknown sp. 36* | na | New York, USA | 1 | - |
|  | *unknown sp. 37* | na | Panama | 1 | - |
|  | *unknown sp. 38* | na | Panama | 1 | - |
|  | *unknown sp. 39* | na | Papua New Guinea | 1 | - |
|  | *unknown sp. 40* | na | California, USA | 1 | - |
|  | *unknown sp. 41* | na | California, USA | 1 | - |
|  | *unknown sp. 42* | na | California, USA | 1 | - |
|  | *unknown sp. 43* | na | California, USA | 1 | - |
|  | *unknown sp. 44* | na | California, USA | 1 | - |
|  | *Anthomyiidae sp. 1* | na | New York, USA | 1 | - |
|  | *Anthomyiidae sp. 2* | na | New York, USA | 1 | - |
|  | *Anthomyiidae sp. 3* | na | Mexico | 1 | - |
|  | *Anthomyiidae sp. 4* | na | Mexico | 1 | - |
|  | *Asilidae sp. 1* | na | New York, USA | 1 | - |
|  | *Asilidae sp. 2* | na | New York, USA | 1 | - |
|  | *Asilidae sp. 3* | na | Mexico | 1 | - |
|  | *Asilidae sp. 4* | na | Mexico | 1 | - |
|  | *Bombyliidae sp.1* | na | Mexico | 1 | - |
|  | *Bombyliidae sp.2* | na | Mexico | 1 | - |
|  | *Bombyliidae sp.3* | na | Mexico | 1 | - |
|  | *Bombyliidae sp.4* | na | Mexico | 1 | + |
|  | *Bombyliidae sp.5* | na | Mexico | 1 | - |
|  | *Bombyliidae sp.6* | na | Mexico | 1 | - |
|  | *Bombyliidae sp.7* | na | Mexico | 1 | - |
|  | *Bombyliidae sp.8* | na | Mexico | 1 | + |
|  | *Bombyliidae sp.9* | na | Mexico | 1 | - |
|  | *Calliphoridae sp. 1* | na | Ghana | 1 | - |
|  | *Calliphoridae sp. 2* | na | Ghana | 1 | - |
|  | *Cecidomyiidae sp. 1* | na | Ghana | 1 | - |
|  | *Cecidomyiidae sp. 2* | na | Ghana | 1 | - |
|  | *Cecidomyiidae sp. 3* | na | Mexico | 1 | - |
|  | *Ceratopogonidae sp. 1* | na | Mexico | 1 | - |
|  | *Ceratopogonidae sp. 2* | na | Mexico | 1 | - |
|  | *Chironomidae sp. 1* | na | Mexico | 1 | - |
|  | *Chironomidae sp. 2* | na | Mexico | 1 | - |
|  | *Chironomidae sp. 3* | na | California, USA | 1 | - |
|  | *Chloropidae sp. 1* | na | Mexico | 1 | - |
|  | *Chloropidae sp. 2* | na | Mexico | 1 | - |
|  | *Chloropidae sp. 3* | na | Mexico | 1 | - |
|  | *Chloropidae sp. 4* | na | Mexico | 1 | - |
|  | *Chloropidae sp. 5* | na | Mexico | 1 | - |
|  | *Chloropidae sp. 6* | na | Mexico | 1 | - |
|  | *Chloropidae sp. 7* | na | Mexico | 1 | - |
|  | *Chloropidae sp. 8* | na | Mexico | 1 | - |
|  | *Chloropidae sp. 9* | na | Mexico | 1 | - |
|  | *Chloropidae sp. 10* | na | Ghana | 1 | - |
|  | *Chloropidae sp. 11* | na | New York, USA | 1 | - |
|  | *Conopidae* | na | Mexico | 1 | - |
|  | *Culicidae sp. 1* | na | New York, USA | 1 | - |
|  | *Culicidae sp. 2* | na | New York, USA | 1 | - |
|  | *Culicidae sp. 3* | na | Michigan | 1 | - |
|  | *Culicidae sp. 4* | na | Mexico | 1 | - |
|  | *Culicidae sp. 5* | na | Mexico | 1 | - |
|  | *Culicidae sp. 6* | na | Mexico | 1 | - |
|  | *Culicidae sp. 7* | na | Mexico | 1 | - |
|  | *Culicidae sp. 8* | na | California, USA | 1 | - |
|  | *Dolichopodidae sp. 1* | na | New York, USA | 1 | - |
|  | *Dolichopodidae sp. 2* | na | New York, USA | 1 | - |
|  | *Dolichopodidae sp. 3* | na | New York, USA | 1 | - |
|  | *Dolichopodidae sp. 4* | na | New York, USA | 1 | - |
|  | *Dolichopodidae sp. 5* | na | New York, USA | 1 | - |
|  | *Dolichopodidae sp. 6* | na | Mexico | 1 | - |
|  | *Dolichopodidae sp. 7* | na | Mexico | 1 | - |
|  | *Dolichopodidae sp. 8* | na | Mexico | 1 | - |
|  | *Dolichopodidae sp. 9* | na | Mexico | 1 | - |
|  | *Dolichopodidae sp. 10* | na | Mexico | 1 | - |
|  | *Dolichopodidae sp. 11* | na | Mexico | 1 | - |
|  | *Dolichopodidae sp. 12* | na | Mexico | 1 | - |
|  | *Dolichopodidae sp. 13* | na | South Africa | 1 | - |
|  | *Drosophilidae sp. 1* | na | Chile | 1 | - |
|  | *Drosophilidae sp. 2* | na | South Africa | 1 | - |
|  | *Drosophilidae sp. 3* | na | South Africa | 1 | - |
|  | *Empididae sp. 1* | na | Mexico | 1 | - |
|  | *Empididae sp. 2* | na | Mexico | 1 | - |
|  | *Ephydridae sp. 1* | na | Mexico | 1 | - |
|  | *Ephydridae sp. 2* | na | Mexico | 1 | - |
|  | *Haliplidae* | na | Mexico | 1 | - |
|  | *Lauxaniidae sp. 1* | na | Mexico | 1 | - |
|  | *Lauxaniidae sp. 2* | na | Mexico | 1 | - |
|  | *Longchopteridae sp. 1* | na | New York, USA | 1 | - |
|  | *Longchopteridae sp. 2* | na | New York, USA | 1 | - |
|  | *Longchopteridae sp. 3* | na | New York, USA | 1 | - |
|  | *Muscidae sp. 1* | na | Mexico | 1 | - |
|  | *Muscidae sp. 2* | na | Mexico | 1 | - |
|  | *Muscidae sp. 3* | na | Mexico | 1 | - |
|  | *Muscidae sp. 4* | na | Mexico | 1 | - |
|  | *Muscidae sp. 5* | na | Mexico | 1 | - |
|  | *Muscidae sp. 6* | na | Mexico | 1 | - |
|  | *Muscidae sp. 7* | na | Mexico | 1 | - |
|  | *Muscidae sp. 8* | na | Mexico | 1 | - |
|  | *Muscidae sp. 9* | na | Mexico | 1 | - |
|  | *Muscidae sp. 10* | na | Mexico | 1 | - |
|  | *Muscidae sp. 11* | na | Chile | 1 | - |
|  | *Muscidae sp. 12* | na | Chile | 1 | - |
|  | *Muscidae sp. 13* | na | Michigan | 1 | - |
|  | *Muscidae sp. 14* | na | California, USA | 1 | - |
|  | *Phoridae sp. 1* | na | Mexico | 1 | - |
|  | *Phoridae sp. 2* | na | Mexico | 1 | - |
|  | *Phoridae sp. 3* | na | Mexico | 1 | - |
|  | *Phoridae sp. 4* | na | Mexico | 1 | - |
|  | *Phoridae sp. 5* | na | Mexico | 1 | - |
|  | *Phoridae sp. 6* | na | Mexico | 1 | - |
|  | *Phoridae sp. 7* | na | Mexico | 1 | - |
|  | *Phoridae sp. 8* | na | Ghana | 1 | - |
|  | *Phoridae sp. 9* | na | Chile | 1 | - |
|  | *Phoridae sp. 10* | na | Chile | 1 | - |
|  | *Phoridae sp. 11* | na | South Africa | 1 | - |
|  | *Phoridae sp. 12* | na | South Africa | 1 | - |
|  | *Phoridae sp. 13* | na | South Africa | 1 | - |
|  | *Pipunculidae* | na | Michigan | 1 | - |
|  | *Platypezidae* | na | New York, USA | 1 | - |
|  | *Platystomatidae* | na | Mexico | 1 | - |
|  | *Psilidae sp. 1* | na | Mexico | 1 | - |
|  | *Psilidae sp. 2* | na | Mexico | 1 | - |
|  | *Sarcophagidae* | na | Mexico | 1 | - |
|  | *Scatopsidae* | na | Mexico | 1 | - |
|  | *Sciaridae sp. 1* | na | New York, USA | 1 | - |
|  | *Sciaridae sp. 2* | na | Mexico | 1 | - |
|  | *Sciaridae sp. 3* | na | Mexico | 1 | - |
|  | *Sciomyzidae sp. 1* | na | New York, USA | 1 | - |
|  | *Sciomyzidae sp. 2* | na | New York, USA | 1 | - |
|  | *Sciomyzidae sp. 3* | na | New York, USA | 1 | - |
|  | *Sciomyzidae sp. 4* | na | New York, USA | 1 | - |
|  | *Sepsidae sp. 1* | na | New York, USA | 1 | - |
|  | *Sepsidae sp. 2* | na | New York, USA | 1 | - |
|  | *Simuliidae sp. 1* | na | Ghana | 1 | - |
|  | *Simuliidae sp. 2* | na | Ghana | 1 | - |
|  | *Simuliidae sp. 3* | na | Mexico | 1 | - |
|  | *Sphaeroceridae sp. 1* | na | New York, USA | 1 | - |
|  | *Sphaeroceridae sp. 2* | na | Mexico | 1 | - |
|  | *Stratiomyidae sp. 1* | na | Mexico | 1 | - |
|  | *Stratiomyidae sp. 2* | na | Mexico | 1 | - |
|  | *Syrphidae sp. 1* | na | New York, USA | 1 | - |
|  | *Syrphidae sp. 2* | na | New York, USA | 1 | - |
|  | *Syrphidae sp. 3* | na | New York, USA | 1 | - |
|  | *Syrphidae sp. 4* | na | New York, USA | 1 | - |
|  | *Syrphidae sp. 5* | na | New York, USA | 1 | - |
|  | *Syrphidae sp. 6* | na | Mexico | 1 | - |
|  | *Syrphidae sp. 7* | na | Mexico | 1 | - |
|  | *Syrphidae sp. 8* | na | Mexico | 1 | - |
|  | *Syrphidae sp. 9* | na | Mexico | 1 | - |
|  | *Syrphidae sp. 10* | na | Mexico | 1 | - |
|  | *Syrphidae sp. 11* | na | Mexico | 1 | - |
|  | *Syrphidae sp. 12* | na | Mexico | 1 | - |
|  | *Syrphidae sp. 13* | na | Mexico | 1 | - |
|  | *Syrphidae sp. 14* | na | Mexico | 1 | - |
|  | *Syrphidae sp. 15* | na | Michigan | 1 | - |
|  | *Tabanidae sp. 1* | na | New York, USA | 1 | - |
|  | *Tabanidae sp. 2* | na | New York, USA | 1 | - |
|  | *Tabanidae sp. 3* | na | New York, USA | 1 | - |
|  | *Tabanidae sp. 4* | na | Mexico | 1 | - |
|  | *Tachinidae sp. 1* | na | Mexico | 1 | - |
|  | *Tachinidae sp. 2* | na | Mexico | 1 | - |
|  | *Tachinidae sp. 3* | na | Mexico | 1 | - |
|  | *Tachinidae sp. 4* | na | Mexico | 1 | - |
|  | *Tachinidae sp. 5* | na | Mexico | 1 | - |
|  | *Tachinidae sp. 6* | na | Mexico | 1 | - |
|  | *Tachinidae sp. 7* | na | Michigan | 1 | - |
|  | *Tachinidae sp. 8* | na | Michigan | 1 | - |
|  | *Tachinidae sp. 9* | na | Michigan | 1 | - |
|  | *Tachinidae sp. 10* | na | Ghana | 1 | - |
|  | *Tephritidae sp. 1* | na | Mexico | 1 | - |
|  | *Tephritidae sp. 2* | na | Mexico | 1 | - |
|  | *Tephritidae sp. 3* | na | Mexico | 1 | - |
|  | *Tephritidae sp. 4* | na | Mexico | 1 | - |
|  | *Tephritidae sp. 5* | na | Mexico | 1 | - |
|  | *Tephritidae sp. 6* | na | Mexico | 1 | - |
|  | *Tephritidae sp. 7* | na | Mexico | 1 | - |
|  | *Tephritidae sp. 8* | na | Mexico | 1 | - |
|  | *Tephritidae sp. 9* | na | Mexico | 1 | - |
|  | *Tephritidae sp. 10* | na | Mexico | 1 | - |
|  | *Therevidae sp. 1* | na | Michigan | 1 | - |
|  | *Therevidae sp. 2* | na | Chile | 1 | - |
|  | *Tipulidae sp. 1* | na | New York, USA | 1 | - |
|  | *Tipulidae sp. 2* | na | New York, USA | 1 | - |
|  | *Tipulidae sp. 3* | na | New York, USA | 1 | - |
|  | *Tipulidae sp. 4* | na | New York, USA | 1 | - |
|  | *Tipulidae sp. 5* | na | Ghana | 1 | - |
|  | *Ulidiidae* | na | Mexico | 1 | - |
| Hemiptera | *unknown sp. 1* | na | unknown | 1 | - |
|  | *unknown sp. 2* | na | Mexico | 1 | - |
|  | *unknown sp. 3* | na | New York, USA | 1 | - |
|  | *unknown sp. 4* | na | Mexico | 1 | - |
|  | *unknown sp. 5* | na | Mexico | 1 | - |
|  | *unknown sp. 6* | na | Mexico | 1 | - |
|  | *unknown sp. 7* | na | Mexico | 1 | - |
|  | *unknown sp. 8* | na | Mexico | 1 | - |
|  | *unknown sp. 9* | na | Mexico | 1 | - |
|  | *unknown sp. 10* | na | Mexico | 1 | - |
|  | *unknown sp. 11* | na | Mexico | 1 | - |
|  | *unknown sp. 12* | na | Mexico | 1 | - |
|  | *unknown sp. 13* | na | Mexico | 1 | - |
|  | *unknown sp. 14* | na | Mexico | 1 | - |
|  | *unknown sp. 15* | na | Mexico | 1 | - |
|  | *unknown sp. 16* | na | Mexico | 1 | - |
|  | *unknown sp. 17* | na | Mexico | 1 | - |
|  | *unknown sp. 18* | na | Mexico | 1 | - |
|  | *unknown sp. 19* | na | Mexico | 1 | - |
|  | *unknown sp. 20* | na | Mexico | 1 | - |
|  | *unknown sp. 21* | na | Panama | 1 | - |
|  | *unknown sp. 22* | na | Panama | 1 | - |
|  | *unknown sp. 23* | na | Panama | 1 | - |
|  | *Aphididae sp. 1* | na | Chile | 1 | - |
|  | *Aphididae sp. 2* | na | South Africa | 1 | - |
|  | *Aphididae sp. 3* | na | Chile | 1 | - |
|  | *Cercopidae* | na | Ghana |  | + |
|  | *Cicadellidae sp. 1* | na | Mexico | 1 | - |
|  | *Cicadellidae sp. 2* | na | Mexico | 1 | - |
|  | *Cicadellidae sp. 3* | na | Chile | 1 | - |
|  | *Cicadellidae sp. 4* | na | South Africa | 1 | - |
|  | *Cicadellidae sp. 5* | na | South Africa | 1 | - |
|  | *Cicadellidae sp. 6* | na | Chile | 1 | - |
|  | *Cicadellidae sp. 7* | na | Chile | 1 | - |
|  | *Cicadellidae sp. 8* | na | Chile | 1 | - |
|  | *Cicadellidae sp. 9* | na | Chile | 1 | - |
|  | *Cicadellidae sp. 10* | na | Panama | 1 | - |
|  | *Cicadellidae sp. 11* | na | Panama | 1 | - |
|  | *Cicadellidae sp. 12* | na | Panama | 1 | - |
|  | *Cicadellidae sp. 13* | na | Panama | 1 | - |
|  | *Cicadellidae sp. 14* | na | Panama | 1 | - |
|  | *Cicadellidae sp. 15* | na | Panama | 1 | - |
|  | *Cicadellidae sp. 16* | na | Panama | 1 | - |
|  | *Cicadellidae sp. 17* | na | Panama | 1 | - |
|  | *Cicadellidae sp. 18* | na | Panama | 1 | - |
|  | *Cicadellidae sp. 19* | na | Panama | 1 | - |
|  | *Cicadellidae sp. 20* | na | Panama | 1 | - |
|  | *Cicadellidae sp. 21* | na | Papua New Guinea | 1 | - |
|  | *Cicadidae sp. 1* | na | Ghana | 1 | - |
|  | *Cicadidae sp. 2* | na | Ghana | 1 | - |
|  | *Coreidae* | na | Papua New Guinea | 1 | - |
|  | *Cydnidae sp. 1* | na | India | 1 | - |
|  | *Cydnidae sp. 2* | na | India | 1 | - |
|  | *Cydnidae sp. 3* | na | India | 1 | - |
|  | *Cydnidae sp. 4* | na | India | 1 | - |
|  | *Delphacidae* | na | India | 1 | - |
|  | *Derbidae sp. 1* | na | Papua New Guinea | 1 | - |
|  | *Derbidae sp. 2* | na | Papua New Guinea | 1 | - |
|  | *Fugoridae sp. 1* | na | Panama | 1 | - |
|  | *Fugoridae sp. 2* | na | Panama | 1 | - |
|  | *Fugoridae sp. 3* | na | Panama | 1 | - |
|  | *Fugoridae sp. 4* | na | Papua New Guinea | 1 | - |
|  | *Gelastocoridae* | na | Ghana | 1 | - |
|  | *Lygaeidae* | na | Papua New Guinea | 1 | - |
|  | *Membracidae sp. 1* | na | Mexico | 1 | - |
|  | *Membracidae sp. 2* | na | Mexico | 1 | - |
|  | *Membracidae sp. 3* | na | Mexico | 1 | - |
|  | *Membracidae sp. 4* | na | Mexico | 1 | - |
|  | *Membracidae sp. 5* | na | Mexico | 1 | - |
|  | *Membracidae sp. 6* | na | Mexico | 1 | - |
|  | *Membracidae sp. 7* | na | Panama | 1 | - |
|  | *Miridae sp. 1* | na | Mexico | 1 | - |
|  | *Miridae sp. 2* | na | South Africa | 1 | - |
|  | *Miridae sp. 3* | na | South Africa | 1 | - |
|  | *Nepidae* | na | Ghana | 1 | - |
|  | *Pentatomidae sp. 1* | na | Mexico | 1 | - |
|  | *Pentatomidae sp. 2* | na | Mexico | 1 | - |
|  | *Pentatomidae sp. 3* | na | Ghana | 1 | - |
|  | *Pentatomidae sp. 4* | na | Mexico | 1 | - |
|  | *Pentatomidae sp. 5* | na | India | 1 | - |
|  | *Pentatomidae sp. 6* | na | Chile | 1 | - |
|  | *Pentatomidae sp. 7* | na | New York, USA | 1 | - |
|  | *Pentatomidae sp. 8* | na | Panama | 1 | - |
|  | *Pentatomidae sp. 9* | na | Papua New Guinea | 1 | - |
|  | *Pyrrhocoridae* | na | Ghana | 1 | - |
|  | *Reduviidae sp. 1* | na | Mexico | 1 | - |
|  | *Reduviidae sp. 2* | na | Ghana | 1 | - |
|  | *Reduviidae sp. 3* | na | Ghana | 1 | - |
|  | *Reduviidae sp. 4* | na | Ghana | 1 | - |
|  | *Reduviidae sp. 5* | na | Mexico | 1 | - |
|  | *Reduviidae sp. 6* | na | New York, USA | 1 | - |
|  | *Reduviidae sp. 7* | na | Panama | 1 | + |
|  | *Ricaniidae* | na | Papua New Guinea | 1 | - |
|  | *Saldidae* | na | India | 1 | - |
| Hymenoptera | *unknown sp. 1* | na | Spain | 1 | - |
|  | *unknown sp. 2* | na | unknown | 1 | - |
|  | *unknown sp. 3* | na | Mexico | 1 | - |
|  | *unknown sp. 4* | na | Mexico | 1 | - |
|  | *unknown sp. 5* | na | Mexico | 1 | - |
|  | *unknown sp. 6* | na | Mexico | 1 | - |
|  | *unknown sp. 7* | na | Mexico | 1 | - |
|  | *unknown sp. 8* | na | Mexico | 1 | - |
|  | *unknown sp. 9* | na | Mexico | 1 | - |
|  | *unknown sp. 10* | na | Mexico | 1 | - |
|  | *unknown sp. 11* | na | Mexico | 1 | - |
|  | *unknown sp. 12* | na | Mexico | 1 | - |
|  | *unknown sp. 13* | na | Mexico | 1 | - |
|  | *unknown sp. 14* | na | Mexico | 1 | - |
|  | *unknown sp. 15* | na | Mexico | 1 | - |
|  | *unknown sp. 16* | na | Mexico | 1 | - |
|  | *unknown sp. 17* | na | Mexico | 1 | - |
|  | *unknown sp. 18* | na | Mexico | 1 | - |
|  | *unknown sp. 19* | na | Mexico | 1 | - |
|  | *unknown sp. 20* | na | Mexico | 1 | - |
|  | *unknown sp. 21* | na | Mexico | 1 | - |
|  | *unknown sp. 22* | na | South Africa | 1 | - |
|  | *Andrenidae sp. 1* | na | Mexico | 1 | - |
|  | *Andrenidae sp. 2* | na | Mexico | 1 | - |
|  | *Andrenidae sp. 3* | na | Mexico | 1 | - |
|  | *Andrenidae sp. 4* | na | Mexico | 1 | - |
|  | *Andrenidae sp. 5* | na | Mexico | 1 | - |
|  | *Andrenidae sp. 6* | na | Mexico | 1 | - |
|  | *Andrenidae sp. 7* | na | Mexico | 1 | - |
|  | *Andrenidae sp. 8* | na | Mexico | 1 | - |
|  | *Andrenidae sp. 9* | na | Mexico | 1 | - |
|  | *Andrenidae sp. 10* | na | Mexico | 1 | - |
|  | *Andrenidae sp. 11* | na | Mexico | 1 | - |
|  | *Andrenidae sp. 12* | na | Mexico | 1 | - |
|  | *Andrenidae sp. 13* | na | Mexico | 1 | - |
|  | *Andrenidae sp. 14* | na | Mexico | 1 | - |
|  | *Apidae sp. 1* | na | India | 1 | - |
|  | *Apidae sp. 2* | na | Mexico | 1 | - |
|  | *Apidae sp. 3* | na | Mexico | 1 | - |
|  | *Apidae sp. 4* | na | Mexico | 1 | - |
|  | *Apidae sp. 5* | na | Mexico | 1 | - |
|  | *Apidae sp. 6* | na | Mexico | 1 | - |
|  | *Apidae sp. 7* | na | Mexico | 1 | - |
|  | *Apidae sp. 8* | na | Mexico | 1 | - |
|  | *Apidae sp. 9* | na | Mexico | 1 | - |
|  | *Apidae sp. 10* | na | Mexico | 1 | - |
|  | *Apidae sp. 11* | na | Mexico | 1 | - |
|  | *Apidae sp. 12* | na | Mexico | 1 | - |
|  | *Apidae sp. 13* | na | Mexico | 1 | - |
|  | *Apidae sp. 14* | na | Mexico | 1 | - |
|  | *Apidae sp. 15* | na | Mexico | 1 | - |
|  | *Apidae sp. 16* | na | New York, USA | 1 | - |
|  | *Apidae sp. 17* | na | New York, USA | 1 | - |
|  | *Apidae sp. 18* | na | New York, USA | 1 | - |
|  | *Apidae sp. 19* | na | New York, USA | 1 | - |
|  | *Apidae sp. 20* | na | California, USA | 1 | - |
|  | *Braconidae sp. 1* | na | Mexico | 1 | - |
|  | *Braconidae sp. 2* | na | Mexico | 1 | - |
|  | *Chalcidae sp. 1* | na | Mexico | 1 | - |
|  | *Chalcidae sp. 2* | na | Mexico | 1 | - |
|  | *Chalcidae sp. 3* | na | Mexico | 1 | - |
|  | *Chalcidae sp. 4* | na | Mexico | 1 | - |
|  | *Chalcidae sp. 5* | na | Mexico | 1 | - |
|  | *Chalcidae sp. 6* | na | Mexico | 1 | - |
|  | *Chalcidae sp. 7* | na | Mexico | 1 | - |
|  | *Chalcidae sp. 8* | na | Mexico | 1 | - |
|  | *Chalcidae sp. 9* | na | Mexico | 1 | - |
|  | *Chalcidae sp. 10* | na | Mexico | 1 | - |
|  | *Chalcidae sp. 11* | na | Mexico | 1 | - |
|  | *Chalcidae sp. 12* | na | Mexico | 1 | - |
|  | *Chalcidae sp. 13* | na | Mexico | 1 | - |
|  | *Chrysididae sp. 1* | na | Mexico | 1 | - |
|  | *Chrysididae sp. 2* | na | Mexico | 1 | - |
|  | *Colletidae sp. 1* | na | Mexico | 1 | - |
|  | *Colletidae sp. 2* | na | Mexico | 1 | - |
|  | *Colletidae sp. 3* | na | Mexico | 1 | - |
|  | *Crabronidae sp. 1* | na | Mexico | 1 | - |
|  | *Crabronidae sp. 2* | na | Mexico | 1 | - |
|  | *Crabronidae sp. 3* | na | Mexico | 1 | - |
|  | *Crabronidae sp. 4* | na | Mexico | 1 | - |
|  | *Crabronidae sp. 5* | na | Mexico | 1 | - |
|  | *Crabronidae sp. 6* | na | Mexico | 1 | - |
|  | *Crabronidae sp. 7* | na | Mexico | 1 | - |
|  | *Crabronidae sp. 8* | na | Mexico | 1 | - |
|  | *Crabronidae sp. 9* | na | Mexico | 1 | - |
|  | *Crabronidae sp. 10* | na | Mexico | 1 | - |
|  | *Crabronidae sp. 11* | na | Mexico | 1 | - |
|  | *Crabronidae sp. 12* | na | Mexico | 1 | - |
|  | *Crabronidae sp. 13* | na | Mexico | 1 | - |
|  | *Crabronidae sp. 14* | na | Mexico | 1 | - |
|  | *Crabronidae sp. 15* | na | Mexico | 1 | - |
|  | *Crabronidae sp. 16* | na | Mexico | 1 | - |
|  | *Crabronidae sp. 17* | na | Mexico | 1 | - |
|  | *Crabronidae sp. 18* | na | Mexico | 1 | - |
|  | *Diapriidae sp. 1* | na | India | 1 | - |
|  | *Diapriidae sp. 2* | na | India | 1 | - |
|  | *Eurytomidae* | na | Mexico | 1 | - |
|  | *Figitidae* | na | New York, USA | 1 | - |
|  | *Formicidae sp. 1* | na | Ghana | 1 | - |
|  | *Formicidae sp. 2* | na | India | 1 | - |
|  | *Formicidae sp. 3* | na | India | 1 | - |
|  | *Formicidae sp. 4* | na | India | 1 | - |
|  | *Formicidae sp. 5* | na | India | 1 | - |
|  | *Formicidae sp. 6* | na | Mexico | 1 | - |
|  | *Formicidae sp. 7* | na | Mexico | 1 | - |
|  | *Formicidae sp. 8* | na | Mexico | 1 | - |
|  | *Formicidae sp. 9* | na | Mexico | 1 | - |
|  | *Formicidae sp. 10* | na | Mexico | 1 | - |
|  | *Formicidae sp. 11* | na | Mexico | 1 | - |
|  | *Formicidae sp. 12* | na | Mexico | 1 | - |
|  | *Formicidae sp. 13* | na | Mexico | 1 | - |
|  | *Formicidae sp. 14* | na | Mexico | 1 | - |
|  | *Formicidae sp. 15* | na | Mexico | 1 | - |
|  | *Formicidae sp. 16* | na | Mexico | 1 | - |
|  | *Formicidae sp. 17* | na | Mexico | 1 | - |
|  | *Formicidae sp. 18* | na | Mexico | 1 | - |
|  | *Formicidae sp. 19* | na | Mexico | 1 | - |
|  | *Formicidae sp. 20* | na | Mexico | 1 | - |
|  | *Formicidae sp. 21* | na | Mexico | 1 | - |
|  | *Formicidae sp. 22* | na | Mexico | 1 | - |
|  | *Formicidae sp. 23* | na | Papua New Guinea | 1 | - |
|  | *Formicidae sp. 24* | na | Rochester | 1 | - |
|  | *Gasteruptiidae sp. 1* | na | Mexico | 1 | - |
|  | *Gasteruptiidae sp. 2* | na | New York, USA | 1 | - |
|  | *Halictidae sp. 1* | na | Mexico | 1 | - |
|  | *Halictidae sp. 2* | na | Mexico | 1 | - |
|  | *Halictidae sp. 3* | na | Mexico | 1 | - |
|  | *Halictidae sp. 4* | na | Mexico | 1 | - |
|  | *Halictidae sp. 5* | na | Mexico | 1 | - |
|  | *Halictidae sp. 6* | na | Mexico | 1 | - |
|  | *Halictidae sp. 7* | na | Mexico | 1 | - |
|  | *Halictidae sp. 8* | na | Mexico | 1 | - |
|  | *Halictidae sp. 9* | na | Mexico | 1 | - |
|  | *Halictidae sp. 10* | na | Mexico | 1 | - |
|  | *Halictidae sp. 11* | na | Mexico | 1 | - |
|  | *Halictidae sp. 12* | na | Mexico | 1 | - |
|  | *Halictidae sp. 13* | na | Mexico | 1 | - |
|  | *Halictidae sp. 14* | na | Mexico | 1 | - |
|  | *Halictidae sp. 15* | na | Mexico | 1 | - |
|  | *Halictidae sp. 16* | na | Mexico | 1 | - |
|  | *Halictidae sp. 17* | na | Mexico | 1 | - |
|  | *Halictidae sp. 18* | na | Mexico | 1 | - |
|  | *Halictidae sp. 19* | na | Mexico | 1 | - |
|  | *Halictidae sp. 20* | na | Mexico | 1 | - |
|  | *Halictidae sp. 21* | na | Mexico | 1 | - |
|  | *Halictidae sp. 22* | na | Mexico | 1 | - |
|  | *Halictidae sp. 23* | na | Mexico | 1 | - |
|  | *Halictidae sp. 24* | na | Mexico | 1 | - |
|  | *Halictidae sp. 25* | na | New York, USA | 1 | - |
|  | *Halictidae sp. 26* | na | New York, USA | 1 | - |
|  | *Ichneumonidae sp. 1* | na | Mexico | 1 | - |
|  | *Ichneumonidae sp. 2* | na | Mexico | 1 | - |
|  | *Ichneumonidae sp. 3* | na | Mexico | 1 | - |
|  | *Ichneumonidae sp. 4* | na | Mexico | 1 | - |
|  | *Ichneumonidae sp. 5* | na | Mexico | 1 | - |
|  | *Ichneumonidae sp. 6* | na | New York, USA | 1 | - |
|  | *Ichneumonidae sp. 7* | na | Papua New Guinea | 1 | - |
|  | *Leucospidae* | na | Mexico | 1 | - |
|  | *Megachilidae sp. 1* | na | Mexico | 1 | - |
|  | *Megachilidae sp. 2* | na | Mexico | 1 | - |
|  | *Megachilidae sp. 3* | na | Mexico | 1 | - |
|  | *Megachilidae sp. 4* | na | Mexico | 1 | - |
|  | *Megachilidae sp. 5* | na | Mexico | 1 | - |
|  | *Megachilidae sp. 6* | na | California, USA | 1 | - |
|  | *Mutillidae sp 1.* | na | Mexico | 1 | - |
|  | *Mutillidae sp 1.* | na | Mexico | 1 | - |
|  | *Mutillidae sp 1.* | na | Mexico | 1 | - |
|  | *Mutillidae sp 1.* | na | Mexico | 1 | - |
|  | *Mutillidae sp 1.* | na | Mexico | 1 | - |
|  | *Mutillidae sp 1.* | na | Mexico | 1 | - |
|  | *Mutillidae sp 1.* | na | Mexico | 1 | - |
|  | *Mutillidae sp 1.* | na | Mexico | 1 | - |
|  | *Mutillidae sp 1.* | na | Mexico | 1 | - |
|  | *Mutillidae sp 1.* | na | Mexico | 1 | - |
|  | *Mutillidae sp 1.* | na | Mexico | 1 | - |
|  | *Mutillidae sp 1.* | na | Mexico | 1 | - |
|  | *Mutillidae sp 1.* | na | Mexico | 1 | - |
|  | *Mutillidae sp 1.* | na | Mexico | 1 | - |
|  | *Pemphredonidae* | na | Mexico | 1 | - |
|  | *Pompilidae sp. 1* | na | Mexico | 1 | - |
|  | *Pompilidae sp. 2* | na | Mexico | 1 | - |
|  | *Pompilidae sp. 3* | na | Mexico | 1 | - |
|  | *Pompilidae sp. 4* | na | Mexico | 1 | - |
|  | *Pompilidae sp. 5* | na | Mexico | 1 | - |
|  | *Pompilidae sp. 6* | na | Mexico | 1 | - |
|  | *Pompilidae sp. 7* | na | Mexico | 1 | - |
|  | *Pompilidae sp. 8* | na | Mexico | 1 | - |
|  | *Pompilidae sp. 9* | na | Mexico | 1 | - |
|  | *Pompilidae sp. 10* | na | Mexico | 1 | - |
|  | *Pompilidae sp. 11* | na | Mexico | 1 | - |
|  | *Pompilidae sp. 12* | na | Mexico | 1 | - |
|  | *Pompilidae sp. 13* | na | Mexico | 1 | - |
|  | *Pompilidae sp. 14* | na | Mexico | 1 | - |
|  | *Pompilidae sp. 15* | na | Mexico | 1 | - |
|  | *Pompilidae sp. 16* | na | Mexico | 1 | - |
|  | *Pompilidae sp. 17* | na | Mexico | 1 | - |
|  | *Pompilidae sp. 18* | na | Mexico | 1 | - |
|  | *Pteromalidae sp. 1* | na | Florida, USA | 1 | - |
|  | *Pteromalidae sp. 2* | na | Florida, USA | 1 | - |
|  | *Pteromalidae sp. 3* | na | Florida, USA | 1 | - |
|  | *Pteromalidae sp. 4* | na | France | 1 | - |
|  | *Pteromalidae sp. 5* | na | France | 1 | - |
|  | *Pteromalidae sp. 6* | na | Kazakhastan | 1 | - |
|  | *Pteromalidae sp. 7* | na | Mexico | 1 | - |
|  | *Pteromalidae sp. 8* | na | Russia | 1 | - |
|  | *Scoliidae sp. 1* | na | Ghana | 1 | - |
|  | *Scoliidae sp. 2* | na | Ghana | 1 | - |
|  | *Sphecidae sp. 1* | na | Mexico | 1 | - |
|  | *Sphecidae sp. 2* | na | Mexico | 1 | - |
|  | *Sphecidae sp. 3* | na | Mexico | 1 | - |
|  | *Sphecidae sp. 4* | na | Mexico | 1 | - |
|  | *Sphecidae sp. 5* | na | Mexico | 1 | - |
|  | *Sphecidae sp. 6* | na | Mexico | 1 | - |
|  | *Sphecidae sp. 7* | na | Mexico | 1 | - |
|  | *Sphecidae sp. 8* | na | Mexico | 1 | - |
|  | *Sphecidae sp. 9* | na | Mexico | 1 | - |
|  | *Sphecidae sp. 10* | na | Mexico | 1 | - |
|  | *Sphecidae sp. 11* | na | Mexico | 1 | - |
|  | *Sphecidae sp. 12* | na | Mexico | 1 | - |
|  | *Sphecidae sp. 13* | na | Mexico | 1 | - |
|  | *Sphecidae sp. 14* | na | Mexico | 1 | - |
|  | *Sphecidae sp. 15* | na | Mexico | 1 | - |
|  | *Sphecidae sp. 16* | na | Mexico | 1 | - |
|  | *Sphecidae sp. 17* | na | Mexico | 1 | - |
|  | *Sphecidae sp. 18* | na | Mexico | 1 | - |
|  | *Sphecidae sp. 19* | na | Mexico | 1 | - |
|  | *Sphecidae sp. 20* | na | Mexico | 1 | - |
|  | *Sphecidae sp. 21* | na | Mexico | 1 | - |
|  | *Sphecidae sp. 22* | na | Mexico | 1 | - |
|  | *Sphecidae sp. 23* | na | California, USA | 1 | - |
|  | *Tiphiidae sp. 1* | na | Mexico | 1 | - |
|  | *Tiphiidae sp. 2* | na | Mexico | 1 | - |
|  | *Vespidae sp. 1* | na | Mexico | 1 | - |
|  | *Vespidae sp. 2* | na | Mexico | 1 | - |
|  | *Vespidae sp. 3* | na | Mexico | 1 | - |
|  | *Vespidae sp. 4* | na | Mexico | 1 | - |
|  | *Vespidae sp. 5* | na | unknown | 1 | - |
| Lepidoptera | *unknown sp. 1* | na | Chile | 1 | - |
|  | *unknown sp. 2* | na | Chile | 1 | - |
|  | *unknown sp. 3* | na | Mexico | 1 | - |
|  | *unknown sp. 4* | na | Mexico | 1 | - |
|  | *unknown sp. 5* | na | Mexico | 1 | - |
|  | *unknown sp. 6* | na | Mexico | 1 | - |
|  | *unknown sp. 7* | na | Mexico | 1 | - |
|  | *unknown sp. 8* | na | Mexico | 1 | - |
|  | *unknown sp. 9* | na | New York, USA | 1 | - |
|  | *unknown sp. 10* | na | South Africa | 1 | - |
|  | *Arctiidae sp. 1* | na | Ghana | 1 | - |
|  | *Arctiidae sp. 2* | na | Ghana | 1 | - |
|  | *Arctiidae sp. 3* | na | India | 1 | - |
|  | *Arctiidae sp. 4* | na | Mexico | 1 | - |
|  | *Arctiidae sp. 5* | na | New York, USA | 1 | - |
|  | *Geometridae sp. 1* | na | New York, USA | 1 | - |
|  | *Geometridae sp. 2* | na | New York, USA | 1 | - |
|  | *Lycaenidae sp. 1* | na | Mexico | 1 | - |
|  | *Lycaenidae sp. 2* | na | Mexico | 1 | - |
|  | *Lycaenidae sp. 3* | na | New York, USA | 1 | - |
|  | *Lycaenidae sp. 4* | na | New York, USA | 1 | - |
|  | *Noctuidae* | na | Papua New Guinea | 1 | + |
|  | *Nymphalidae sp. 1* | na | Panama | 1 | - |
|  | *Nymphalidae sp. 2* | na | Panama | 1 | - |
|  | *Pieridae* | na | New York, USA | 1 | - |
|  | *Saturniidae* | na | Ghana | 1 | - |
|  | *Mantodea* |  |  |  |  |
|  | *unknown sp.* | na | South Africa | 1 | - |
| Neuroptera | *Chrysopidae sp. 1* | na | Mexico | 1 | + |
|  | *Chrysopidae sp. 2* | na | Mexico | 1 | - |
|  | *Myrmeleontidae sp. 1* | na | Mexico | 1 | - |
|  | *Myrmeleontidae sp. 2* | na | Mexico | 1 | - |
| Odonata | *Coenagrionidae sp.1* | na | Mexico | 1 | - |
|  | *Coenagrionidae sp.2* | na | Mexico | 1 | - |
|  | *Coenagrionidae sp.3* | na | Mexico | 1 | - |
|  | *Coenagrionidae sp.4* | na | Mexico | 1 | - |
|  | *Coenagrionidae sp.5* | na | Mexico | 1 | - |
|  | *Zygopteraidae sp. 1* | na | Spain | 1 | - |
|  | *Zygopteraidae sp. 2* | na | Spain | 1 | - |
|  | *Zygopteraidae sp. 3* | na | Spain | 1 | - |
| Orthoptera | *unknown sp. 1* | na | Spain | 1 | - |
|  | *unknown sp. 2* | na | Spain | 1 | - |
|  | *unknown sp. 3* | na | Mexico | 1 | - |
|  | *Acrididae* | na | Ghana | 1 | - |
|  | *Gryllidae sp. 1* | na | Mexico | 1 | - |
|  | *Gryllidae sp. 2* | na | South Africa | 1 | - |
|  | *Mantidae* | na | Panama | 1 | - |
|  | *Tettigoniidae sp. 1* | na | Panama | 1 | - |
|  | *Tettigoniidae sp. 2* | na | Mexico | 1 | - |
| Psocoptera | *unknown sp.* | na | Chile | 1 | - |
| Siphonaptera | *unknown sp.* | na | Chile | 1 | - |
| Strepsiptera | *unknown sp.* | na | Chile | 1 | - |
| Thysanoptera | *unknown sp. 1* | na | Mexico | 1 | - |
|  | *unknown sp. 2* | na | Chile | 1 | - |
|  | *unknown sp. 3* | na | South Africa | 1 | - |
|  | *unknown sp. 4* | na | South Africa | 1 | - |
|  | *unknown sp. 5* | na | South Africa | 1 | - |
|  | *unknown sp. 6* | na | South Africa | 1 | - |
|  | *unknown sp. 7* | na | South Africa | 1 | - |
|  | *unknown sp. 8* | na | Chile | 1 | - |
|  | *Thripidae* | na | Papua New Guinea | 1 | - |
| Trichoptera | *unknown sp.* | na | South Africa | 1 | - |
